# Widespread disruption of host transcription termination in HSV-1 infection

**DOI:** 10.1038/ncomms8126

**Published:** 2015-05-20

**Authors:** Andrzej J. Rutkowski, Florian Erhard, Anne L’Hernault, Thomas Bonfert, Markus Schilhabel, Colin Crump, Philip Rosenstiel, Stacey Efstathiou, Ralf Zimmer, Caroline C. Friedel, Lars Dölken

**Affiliations:** 1 Division of Infectious Diseases, Department of Medicine, University of Cambridge, Cambridge CB2 0QQ, UK; 2 Institut für Informatik, Ludwig-Maximilians-Universität München, Amalienstraße 17, 80333 München, Germany; 3 Institut für Klinische Molekularbiologie, Christian-Albrechts-Universität Kiel, Schittenhelmstraße 12, 24105 Kiel, Germany; 4 Division of Virology, Department of Pathology, University of Cambridge, Tennis Court Road, Cambridge CB2 1QP, UK; 5 Institut für Virologie, Julius-Maximilians-Universität Würzburg, Versbacher Straße 7, 97078 Würzburg, Germany

## Abstract

Herpes simplex virus 1 (HSV-1) is an important human pathogen and a paradigm for virus-induced host shut-off. Here we show that global changes in transcription and RNA processing and their impact on translation can be analysed in a single experimental setting by applying 4sU-tagging of newly transcribed RNA and ribosome profiling to lytic HSV-1 infection. Unexpectedly, we find that HSV-1 triggers the disruption of transcription termination of cellular, but not viral, genes. This results in extensive transcription for tens of thousands of nucleotides beyond poly(A) sites and into downstream genes, leading to novel intergenic splicing between exons of neighbouring cellular genes. As a consequence, hundreds of cellular genes seem to be transcriptionally induced but are not translated. In contrast to previous reports, we show that HSV-1 does not inhibit co-transcriptional splicing. Our approach thus substantially advances our understanding of HSV-1 biology and establishes HSV-1 as a model system for studying transcription termination.

## Abstract (web summary)

Herpes simplex virus 1 (HSV-1) efficiently shuts down host gene expression in infected cells. Here Rutkowski *et al*. analyse the genome-wide changes in transcription and translation in infected cells, and show that HSV-1 triggers an extensive disruption of transcription termination of cellular genes.

## Graphical abstract

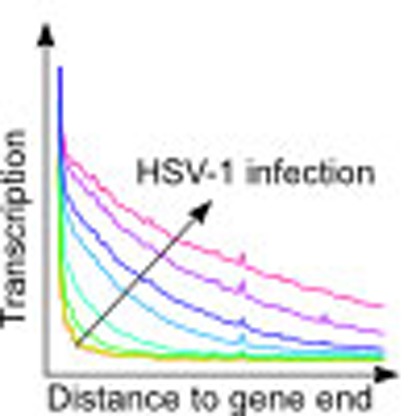

Herpesviruses are large DNA viruses that replicate in the cell nucleus and efficiently take over host cell gene expression machinery during lytic infection. Herpes simplex virus 1 (HSV-1) is one of the eight herpesviruses known to infect humans. It is the causative agent of cold sores but also responsible for life-threatening infections in both immunodeficient and immunocompetent individuals^1^. During lytic infection, HSV-1 rapidly shuts down host gene expression, making it a paradigm for virus-induced ‘host shut-off’. Two viral proteins play a key role in this process. On virus entry, the virion host shut-off (*vhs*) endonuclease, delivered by the incoming virus particles, starts rapidly cleaving both cellular and viral mRNAs^2^,^3^. With the advent of lytic viral gene expression, the viral infected cell polypeptide 27 (*ICP27*) is expressed and thought to contribute to HSV-1 host shut-off by interfering with cellular splicing machinery (reviewed in refs 4, 5, 6). *ICP27* inhibits the splicing of radiolabelled ^32^P-labelled pre-mRNAs *in vitro*^7^,^8^,^9^, interacts with components of the splicing machinery^8^,^10^ and causes a redistribution of splicing factors^11^,^12^. The latter, however, was not sufficient to inhibit host splicing^11^. As only four viral genes contain introns, expression of most viral genes is thought to be unaffected. In addition, HSV-1 reduces the activity of RNA polymerases (RNAPs)^13^,^14^,^15^ and was reported to target RNAP II for proteasomal degradation^16^. Our understanding, however, of how maintenance of vital cell functions and host shut-off are orchestrated remains superficial.

The development of powerful next-generation sequencing techniques now allows capturing of real-time changes in RNA synthesis^17^,^18^,^19^,^20^, processing^21^ and translation^22^,^23^ at whole-transcriptome level. 4sU-tagging (Fig. 1a) utilizes the nucleoside analogue 4-thiouridine (4sU) to metabolically label newly transcribed RNA (4sU-RNA), which can be separated from total cellular RNA by streptavidin affinity purification^17^,^19^,^21^,^24^. RNA sequencing (RNA-seq) of 4sU-RNA (4sU-seq) depicts both real-time transcriptional activity^17^ and the kinetics of RNA processing^21^. In contrast to sequencing total RNA, which to a large degree represents cellular RNA transcribed before infection, 4sU-seq allows studying of real-time transcriptional activity at defined times of infection. Ribosome profiling provides genome-wide quantification of translational activity based on large-scale sequencing of ribosome-protected (that is, actively translated) mRNA fragments (Fig. 1b)^22^,^23^. Ribosome profiling combined with RNA-seq of total RNA has previously been used to annotate the transcriptome and translation products of human cytomegalovirus^23^ and Kaposi’s sarcoma-associated herpesvirus^25^ resulting in the identification of hundreds of new viral gene products. Finally, quantitative analyses of the temporal changes in gene expression using such high-throughput technologies provide insights into the global dynamics of virus–host interaction^26^.

In this study, we combined 4sU-seq and ribosome profiling to study real-time changes in RNA synthesis and processing as well as their impact on translation during HSV-1 lytic infection. Using both technologies, we monitored the full course of lytic HSV-1 infection in human foreskin fibroblasts (HFF) at up to nine time points. We report the surprising observation that HSV-1 disrupts transcription termination of cellular but not viral genes, which is detectable as early as 3–4 h post infection (p.i.). Disrupted transcription termination requires neither *vhs* nor *ICP27.* It leads to extensive intergenic transcription for tens of thousands of nucleotides (nt) beyond polyadenylation (poly(A)) sites and into downstream genes. In contrast to previous reports, we find no evidence that HSV-1 infection results in a generalized inhibition of splicing even in late HSV-1 infection. Instead, transcription from upstream genes into downstream genes and incomplete splicing of the resulting aberrant transcripts explains the observed accumulation of intronic reads. Finally, HSV-1 infection induces aberrant splicing events including both novel intragenic as well as intergenic splicing within the arising multi-gene transcripts.

## Results

### Transcription and translation during HSV-1 infection

**Figure 1:**
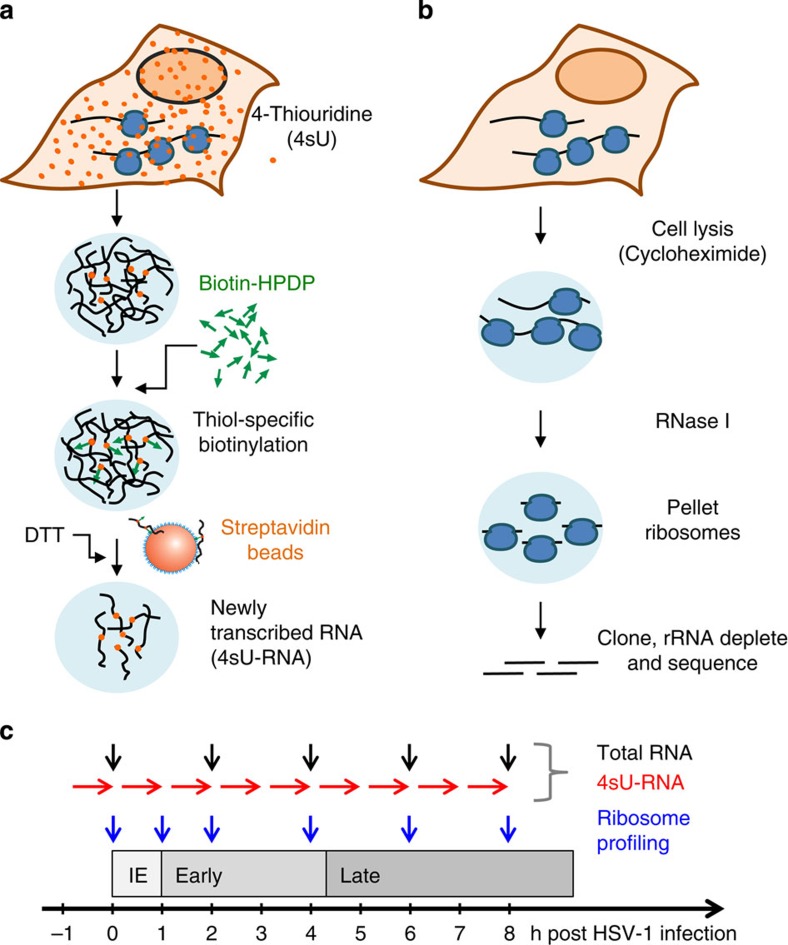
Methodology and experimental design. (**a**) Principle of 4sU-tagging: human fibroblasts are pulse labelled with 4sU. Following isolation of total RNA, newly transcribed RNA (4sU-RNA) is biotinylated at the sulphur atom in 4sU and separated from total RNA using streptavidin-coated magnetic beads. 4sU-RNA is recovered from the beads by adding the reducing agent dithiothreitol (DTT). (**b**) Principle of ribosome profiling: ribosome-protected RNA fragments of 28–32 nucleotides are recovered from RNase-digested ribosome pellets of cytoplasmatic extracts by size selection. cDNA libraries are prepared, rRNA depleted and subjected to next-generation sequencing. (**c**) Experimental set-up: 4sU-tagging was performed in 1 h intervals (indicated by red arrows) during the first 8 h of HSV-1 infection. Extraction of total RNA (black arrows) and ribosome profiling (blue arrows) was performed at the indicated time points.

**Figure 2:**
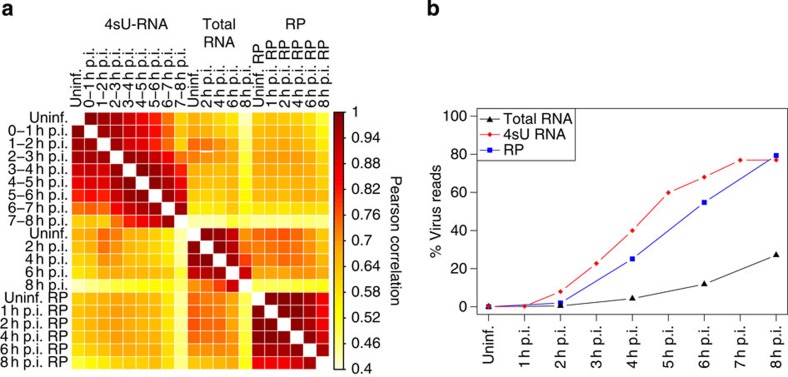
Transcriptional and translational activity during HSV-1 infection. (**a**) Pearson correlation of gene RPKM in 4sU-RNA, total RNA and ribosome profiling (RP). Correlation was calculated across all genes expressed in at least one sample for both RNA-seq and ribosome profiling. RPKM values were averaged between replicates. Translational activity quantified by ribosome profiling correlated better with total RNA than with 4sU-RNA throughout infection. (**b**) Contribution of viral reads to all protein-coding sequence reads in total RNA, 4sU-RNA and ribosome profiling at the indicated time points. For 4sU-RNA, the end of the labelling interval is indicated. This illustrates the efficient takeover of the host cell gene expression machinery by HSV-1.

4sU-tagging was performed in 1 h intervals during the first 8 h of infection (Fig. 1c). We restricted our analyses to the first 8 h of infection as 4sU incorporation into newly transcribed RNA and transcriptional activity remained stable within an approximately twofold range but dropped markedly from 12 to 24 h p.i. (Supplementary Fig. 1). All 4sU-RNA samples, as well as total RNA from every other hour, were subjected to RNA-seq (100 nt paired-end reads). Ribosome profiling was performed at 0, 1, 2, 4, 6 and 8 h p.i. Both assays provided highly reproducible data (two replicates each, correlation between replicates >0.95; Supplementary Fig. 2). This provided a comprehensive view on the temporal changes in RNA synthesis, processing and translation of >10,000 cellular and viral genes (Supplementary Data 1 and 2). Translational activity correlated better with total than with newly transcribed RNA levels, although this correlation decreased at late stages of infection (Fig. 2a). Furthermore, translation rates of viral mRNAs were significantly higher than expected from their prevalence in total RNA, as viral genes accounted for ∼80% of both transcriptional (4sU-RNA) and translational activity (ribosome profiling) on protein-coding sequences at 8 h p.i. (Fig. 2b). In contrast, they only represented 27% of total RNA at this time point. Thus, HSV-1 efficiently usurped the host cell’s gene expression machinery.

**Figure 3:**
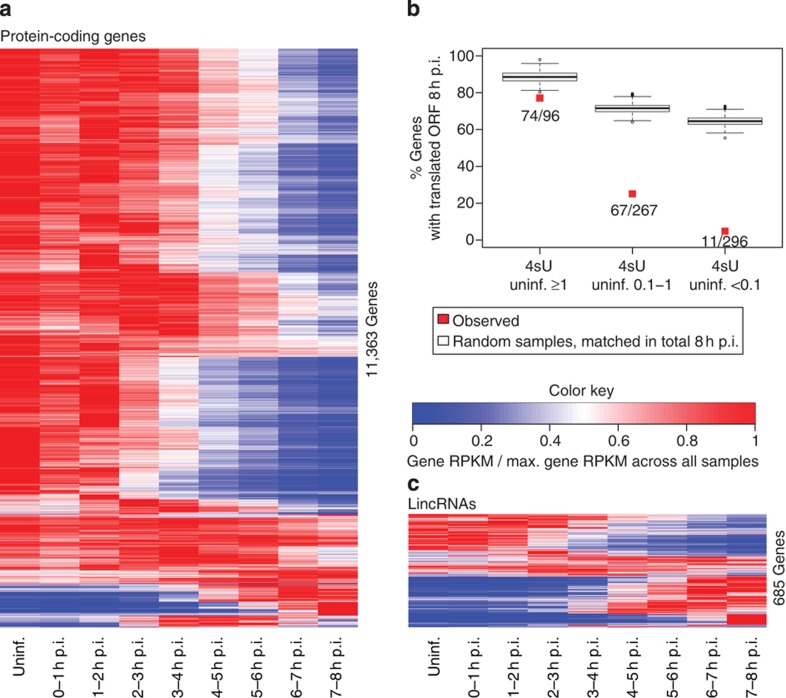
HSV-1-induced host shut-off. (**a**) Heat map of transcriptional changes (4sU-RNA) in 11,363 protein-coding genes. Genes were clustered using hierarchical average linkage clustering and Euclidean distances. For this purpose, RPKM values were normalized to the maximum RPKM across all time points, resulting in values between 0 (=no expression of gene; blue) and 1 (=maximum expression of gene; red). (**b**) Transcriptionally induced genes were grouped according to their RPKM values in 4sU-RNA of uninfected cells and the percentage of genes with translated ORFs at 8 h p.i. were determined for each group. This revealed that most genes induced transcriptionally in HSV-1 infection were not translated. Significance of the results was evaluated by comparing the number of translated ORFs against 1,000 randomly sampled gene sets of non-induced genes matched in number and RPKM values (±0.1) in total RNA at 8 h p.i. (see Methods). The distribution of the percentage of translated ORFs for the randomly sampled non-induced gene sets is depicted as box plots. The boxes indicate the range between the 25th and 75th percentile (=interquartile range (IQR)) around the median (thick horizontal line) of the distribution. The whiskers (=short horizontal lines at ends of dashed vertical line) extend to the data points at most 1.5 × IQR from the box. Data points outside this range are shown as small circles. The observed percentage of translated ORFs was below the smallest value observed for any of the randomly sampled gene sets (*P* value<10^−3^), indicating that their low translation rates were not simply due to low total RNA levels at 8 h p.i. (**c**) Heat map of 685 lincRNAs expressed during HSV-1 infection and clustered as described in **a**.

Lytic HSV-1 infection induced dramatic changes in the transcriptional activity. In 4sU-RNA, 75% of cellular protein-coding genes (8,545 of 11,363) were downregulated, while 659 (5.8%) genes were upregulated (Fig. 3a). Similar changes occurred in total RNA (Supplementary Data 1). Surprisingly, increased transcriptional activity of cellular genes was not matched by a respective increase in translational activity. Only 33 of 9,691 translated genes (0.34%) showed an increased translational activity at 8 h p.i. More importantly, 507 (77%) of the transcriptionally induced protein-coding genes were not translated at all at 8 h p.i., compared with ∼29% of non-induced genes with similar total RNA expression levels (*P*<0.001 as described in the Methods). Interestingly, 485 of 507 genes (96%) with induced transcription but no translation were transcribed poorly or not at all (reads per kilobase per million mapped reads (RPKM) <1, Fig. 3b) in uninfected cells. Finally, a remarkably high number (305 of 685=44.5%) of long intergenic non-coding RNAs (lincRNAs) appeared to be upregulated in 4sU-RNA (Fig. 3c).

### HSV-1 disrupts transcription termination

**Figure 4:**
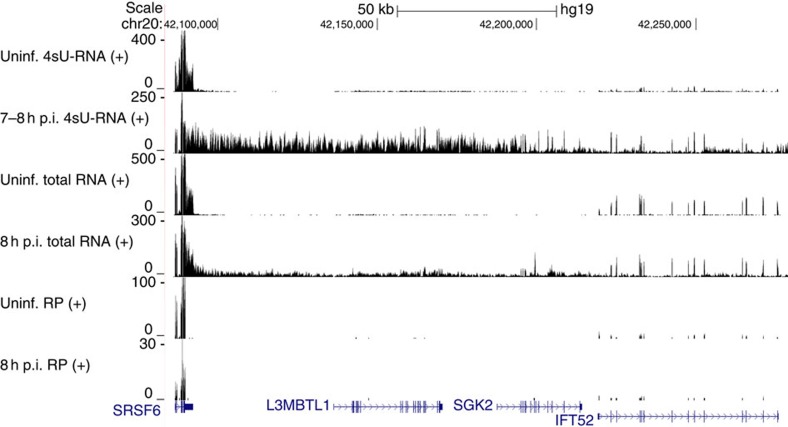
Disruption of transcription termination of the SRSF6 gene. Lytic HSV-1 infection induced massive transcriptional activity downstream of the *SRSF6* gene at 7–8 h p.i. extending into neighbouring genes. Downstream genes were induced sixfold (*L3MBTL1*), 68-fold (*SGK2*) and 2.5-fold (*IFT52*) at 7–8 h p.i., respectively (Supplementary Data 1). Read counts per genome position are shown for 4sU-RNA (row 1 and 2), total RNA (row 3 and 4) and ribosome profiling (RP, row 5 and 6). Ribosome profiling reads to a U6 snRNA repeat at chr20:42,101,653-42,101,758 were masked. For each gene, direction (arrowheads), exons (vertical bars, narrow bars=untranslated sequences) and introns (horizontal lines between bars) are indicated in blue at the bottom.

**Figure 5:**
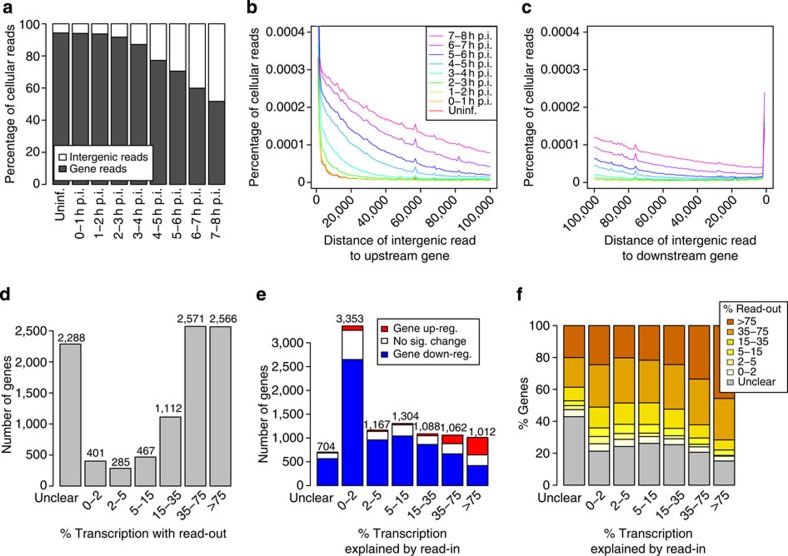
Lytic HSV-1 infection disrupts transcription termination. (**a**) The proportion of 4sU-RNA reads mapping to human genes and intergenic regions illustrates the dramatic increase in intergenic transcription during HSV-1 infection. (**b**,**c**) Distribution of intergenic reads down- (**b**) and upstream (**c**) of genes demonstrates that intergenic transcription peaks at gene 3′-ends and gradually declines with increasing distance from 3′-ends. Only reads within intergenic regions with length ⩾100,000 nt were considered and read counts were determined in 1,000 nt windows downstream of gene 3′-ends and upstream of gene 5′-ends. (**d**) Number of genes with various extents of read-out. Extent of read-out at late times of infection was quantified as % transcription with read-out (=100 × RPKM within the first 5,000 nt downstream of the gene/gene RPKM) at 7–8 h p.i. Read-out (and read-in) was classified as unclear if it was larger than 5% but without significant correlation between downstream (upstream) expression and the course of infection. (**e**) Number of genes with various extents of read-in. Extent of read-in was quantified as % transcription explained by read-in (=100 × RPKM in the first 5,000 nt upstream of the gene/gene RPKM) at 7–8 h p.i. Genes with a high extent of read-in were enriched for upregulated genes (⩾2-fold, see Methods). (**f**) Combined analysis of the extent of read-out and read-in at 7–8 h p.i. Genes with high extent of read-in were also enriched for read-out, that is, even a second poly(A) signal was often not sufficient to terminate transcription.

When analysing genes that were transcriptionally induced but not translated, we observed massive transcriptional activity (4sU-RNA) upstream of their 5′-ends at late times of infection, extending without gaps for tens of thousands of nt to neighbouring upstream genes. Figure 4 shows a typical example where three genes (*L3MBTL1*, *SGK2* and *IFT52*) appear to be induced on transcriptional level (both in total and 4sU-RNA). Only *IFT52*, the only one of these genes transcribed and translated in uninfected cells (RPKM>0.5), is translated during infection but still translationally downregulated (>5-fold). All three genes show extensive upstream transcriptional activity, which originates from the *SRSF6* gene. This indicates that the transcription termination and cleavage machinery is no longer properly recruited or functioning at the *SRSF6* termination signals, resulting in transcription into downstream regions by >100,000 nt (henceforth termed ‘disruption of transcription termination’ and ‘read-out’). Accordingly, reads mapping to intergenic regions increased dramatically starting at 2–3 h p.i. (Fig. 5a). Late in infection, they accounted for ∼50% of all cellular reads in 4sU-RNA. Intergenic transcription peaked at annotated 3′-ends of genes and gradually declined with increasing distance from them (Fig. 5b,c). We excluded the effects of 4sU exposure on transcription termination by performing quantitative reverse transcription–PCR (qRT–PCR) on both 4sU-treated and 4sU-naïve HFF at 8 h p.i. Transcription downstream of expressed genes was equally prominent in presence and absence of 4sU (Supplementary Fig. 3).

To quantify the read-out, we calculated the ratio of RPKM within 5,000 nt downstream of gene 3′-ends to the RPKM of the corresponding gene at 7–8 h p.i. for 9,690 protein-coding and lincRNA genes. By 7–8 h p.i., read-out was >15% of the gene’s RPKM for 64% of cellular genes and >75% for 26% of genes (Fig. 5d). At this time, ∼8,000 cellular genes were still transcribed and ∼6,800 genes translated (RPKM >1 in both cases). Read-out was highly reproducible between replicates (Supplementary Fig. 4a) and also prevalent in total RNA at 8 h p.i. (Fig. 4 and Supplementary Fig. 4b,c). This suggests that the respective transcripts were not grossly unstable. While we observed no functional enrichment among genes with >75% read-out, the extent of read-out correlated with the prevalence of different poly(A) signal sequences within 50 nt of gene 3′-ends. The canonical AAUAAA poly(A) signal was present in 79% of genes with ≤5% read-out, but only in 61% of genes with >35% read-out (*P*<10^−12^, Fisher’s exact test, Supplementary Fig. 4d). In contrast, the weaker AUUAAA signal^27^ was present in 17% genes with >35% read-out compared with 10% genes with ≤5% read-out (*P*<0.00012, Fisher’s exact test). The role of small differences in less well-defined flanking motifs (for example, G/U-rich sequences^28^, Supplementary Fig. 5) remains to be elucidated in further studies.

Late in infection, read-out commonly extended over thousands of nucleotides into downstream genes (denoted as ‘read-in’). At least 32.6% of genes showed read-in >15% of the gene’s RPKM at 7–8 h p.i. (Fig. 5e) and read-in was inversely correlated with the distance to the next upstream gene (Fig. 5b,c; Supplementary Fig. 6). There was no correlation between read-out or read-in and expression levels in 7–8 h p.i. 4sU-RNA (correlation coefficient −0.027 and 0.0096, respectively) as well as expression levels in total RNA at 8 h p.i. (correlation coefficient −0.016 and −0.0008, respectively). Genes with read-in were also enriched for greater read-out (Fig. 5f), indicating that even additional poly(A) signals were often not sufficient to terminate transcription. Only 4.2% of genes (403 of 9,690) showed ≤5% read-out and read-in. These included the housekeeping genes *GAPDH* and *β-actin* (Supplementary Fig. 7) commonly used to analyse RNA processing in HSV-1 infection. This may explain why the defect in transcription termination has been missed to date. Interestingly, the genes with read-out and read-in both ≤5% were on average less strongly downregulated in translation at 6 and 8 h p.i. than genes with read-out >35% and read-in ≤5% (Wilcox rank-sum test *P*<10^−8^) despite there being no corresponding difference in total RNA levels (Supplementary Fig. 8).

For genes with low or no transcription in uninfected cells, read-in is likely to exceed endogenous transcript levels, predisposing it to be misinterpreted as ‘induction’ (Supplementary Fig. 9). Indeed, 36% of genes with >75% read-in appeared to be upregulated in 4sU-RNA, in contrast to ∼2.6% of genes with ≤5% read-in. This was even more pronounced for HSV-1-induced lincRNAs, of which 62% showed >75% read-in, and is explained by their poor expression in HFF^29^,^30^. As a consequence, induction by read-in was also highly prevalent in total RNA (Supplementary Fig. 10) and 405 of 548 (74%) of genes induced by read-in in 4sU-RNA were also induced in total RNA. Furthermore, 397 of 557 cellular genes (71%) that appeared induced in total RNA showed >35% read-in in total RNA. Thus, the extent of read-in needs to be determined when profiling transcriptional activity in HSV-1 infection to avoid misinterpreting the cellular response to HSV-1 infection.

Finally, ribosome profiling demonstrated that read-in generally does not result in functional transcripts as 99% of genes transcribed only due to read-in were not translated (Supplementary Fig. 11). We conclude that read-in, rather than gene promoter activation, is responsible for most of the transcriptional induction during lytic HSV-1 infection.

### Effects of HSV-1 on splicing

**Figure 6:**
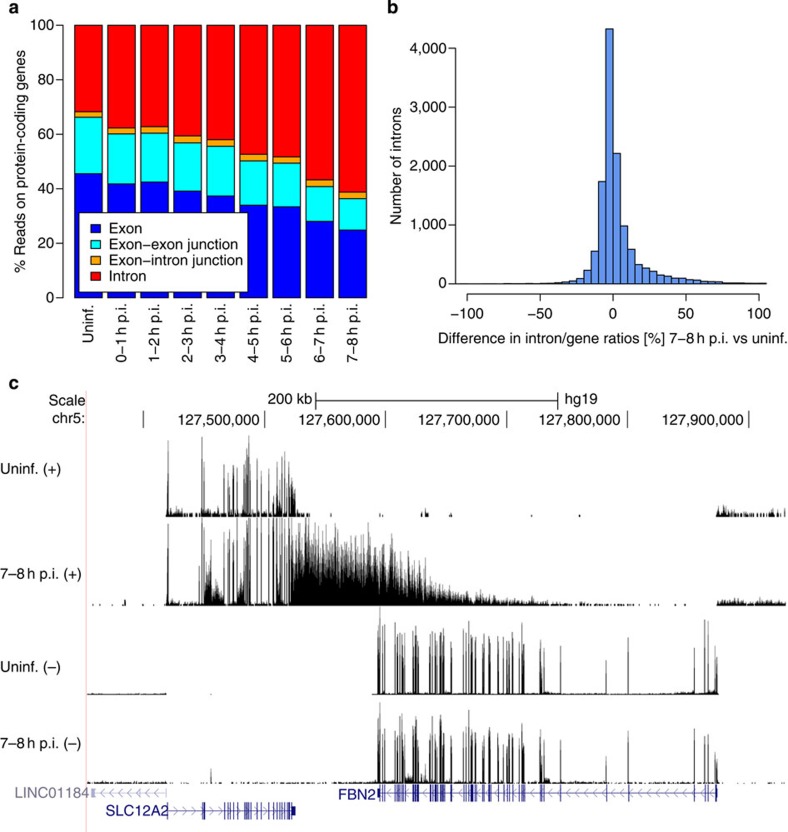
Inhibition of splicing and accumulation of introns in HSV-1 infection. (**a**) Changes in the contribution of intronic, exonic and junction reads for protein-coding genes in 4sU-RNA demonstrate an ∼2-fold increase in intronic sequences during HSV-1 infection. (**b**) Differences of intron/gene ratios (in %) between 7–8 h p.i. and uninfected cells illustrate the changes in the contribution of intron-containing transcripts for ∼2,000 most highly expressed genes. Although the distribution of changes was slightly skewed towards larger increases in intron/gene ratios, no generalized increase in intron/gene ratios occurred during infection. For 56% of introns, intron/gene ratios actually decreased (resulting in negative differences in intron/gene ratios). (**c**) Example of two fully spliced genes (*SLC12A2*, *FBN2*). *SLC12A2* suffers from >75% read-out in 4sU-RNA. Read-out leads to substantial antisense transcription for the *FBN2* gene on the opposite strand, which itself does not suffer from read-out. For each gene, loci are shown in blue at the bottom as described in Fig. 4.

**Figure 7:**
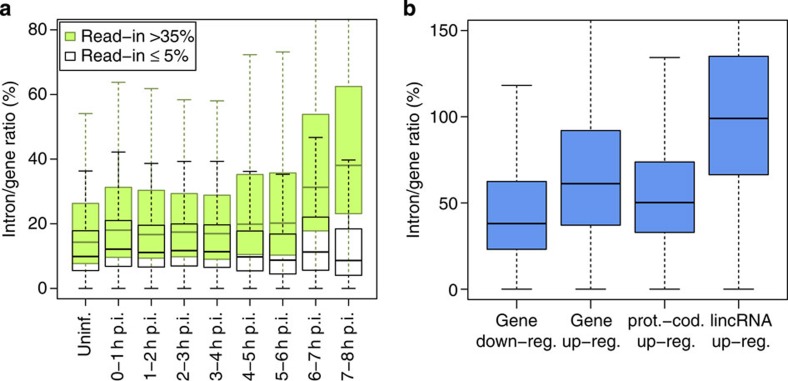
Accumulation of introns in HSV-1 infection is due to read-in. (**a**) Comparison of intron/gene ratios during the course of infection for downregulated genes with and without read-in demonstrates that read-in accounts for the increase in intron/gene ratios. Distributions of intron/gene ratios are depicted as box plots as described in Fig. 3b. For clarity, extreme data points outside the whiskers are not shown. (**b**) Genes transcriptionally induced by read-in (read-in >35%) show even higher intron/gene ratios than downregulated genes with read-in. This increase in intron/gene ratios predominantly derived from lincRNAs, which were generally not spliced at all (indicated by a median intron/gene ratio ∼100%). In contrast, median intron/gene ratios of protein-coding genes were ∼50%, indicating some degree of splicing. Distributions of intron/gene ratios are depicted as box plots as in **a**.

Previous studies reported the inhibition of pre-mRNA splicing by the HSV-1 *ICP27* protein. We thus investigated the change in the contribution of exonic and intronic reads during HSV-1 infection (Fig. 6a). Although we did observe a twofold increase (from 31 to 62%) in intronic reads in 4sU-RNA throughout infection, this is rather modest given that introns are on average ∼30 times longer than exons^31^. To analyse the extent of splicing inhibition for individual genes, we calculated the ratios of intron to gene RPKM in 4sU-RNA for introns of the ∼2,000 most strongly expressed genes. Consistent with the modest increase in intronic reads, we observed no global increase in intron/gene ratios throughout infection (Fig. 6b and Supplementary Fig. 12). Instead, read-in explained the increase in intronic reads (see Fig. 4 for an example), whereas average intron/gene ratios did not increase for genes without read-in (see Fig. 6c for an example and Fig. 7a for a global analysis). This was independent of the extent of read-out (Supplementary Fig. 13). Splicing for all cellular genes is shown in a UCSC Genome Browser session containing 4sU-RNA read density values across the human genome (the session can be accessed at http://www.bio.ifi.lmu.de/HSV-1). This also visualizes read-in, read-out and antisense transcription.

Interestingly, multi-exon lincRNAs induced by read-in were generally not spliced at all (Fig. 7b and Supplementary Fig. 14). In contrast, intron RPKMs for protein-coding genes induced by read-in were only ∼50% of gene RPKMs indicating that most of them were at least partially spliced. Previous studies reported that impaired splicing can result in rapid, co-transcriptional RNA degradation of unspliced or mis-spliced pre-mRNAs^32^. However, overall downregulation of multi-exon genes was not faster than of intron-less genes (Supplementary Fig. 15) arguing against prominent co-transcriptional RNA degradation of mis-spliced transcripts. Therefore, splicing is not generally inhibited in HSV-1 infection. Instead, disruption of transcription termination and read-in is responsible for the observed increase in intron levels. Finally, read-out was also observed for intron-less genes (Supplementary Fig. 16) and, therefore, is not dependent on splicing.

**Figure 8:**
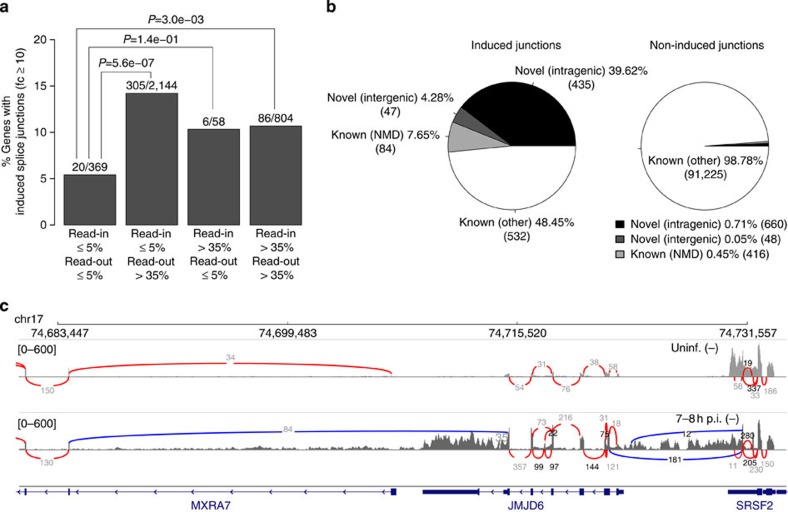
Disruption of transcription termination results in novel splicing events. (**a**) Genes with strong read-out (>35%) but no read-in (≤5%) at 7–8 h p.i. are enriched for strongly induced splice junctions compared with genes with no read-out (≤5%) (*P* values determined with Fisher’s exact test). This indicates that disrupted transcription termination is associated with altered splicing already upstream of the first poly(A) site. (**b**) Comparison of the frequencies of novel intra- and intergenic splicing events and splicing associated with nonsense-mediated decay (NMD) among splice junctions induced during HSV-1 infection (left) and junctions that are not induced (right). Absolute numbers of junctions of each type are in brackets. The increased number of novel and intergenic splicing events among induced junctions hints at abnormal splicing associated with disrupted transcription termination. (**c**) Sashimi plots for two novel intergenic splicing events between the neighbouring *SRSF2*, *JMJD6* and *MXRA7* genes on the negative strand (gene loci depicted in blue as described in Fig. 4). Splice junctions are shown as arcs (red=intragenic, blue=intergenic), with the number of reads mapping to each splice junction annotated to the arcs.

We next investigated whether HSV-1 infection affected splicing in other ways, for example, by inducing abnormal splicing events. We identified 1,098 splice junctions that were induced ⩾10-fold in 7–8 h p.i. 4sU-RNA relative to the fold-change in transcriptional activity of the corresponding gene. In total RNA, we found 668 of these junctions (61%) and 569 of these (85%) were induced ⩾2-fold (48% induced ⩾10-fold). Splice junctions induced in 4sU-RNA were significantly enriched among genes with >35% read-out (Fig. 8a). This indicates that splicing is already affected upstream of poly(A) sites suffering from read-out or that aberrant splicing contributes to the failure in transcription termination of the respective transcripts. Interestingly, 44% of the induced splice junctions were novel, that is, not part of any annotated transcript (Fig. 8b), and 11% of these represented intergenic splicing between exons of two neighbouring genes connected by read-out and subsequent read-in. For example, two novel intergenic splicing events occurred between exons of *SRSF2*, *JMJD6* and *MXRA7* late in infection (Fig. 8c). To confirm that these intergenic splicing events were not an artefact of RNA-seq mapping, we generated a database of all possible intergenic splicing events between annotated exons of gene pairs located ≤100 kb apart (see Methods). We realigned all 4sU-seq reads against this database using the short-read alignment programme BWA^33^. Following a stringent filtering of false positives, we identified 71 intergenic splicing events including the two events between *SRSF2*, *JMJD6* and *MXRA7* (Supplementary Data 3). Of these events, 62 (87%) were exclusively found in 4sU-RNA of infected cells. We also confirmed 52 of these 62 (84%) events in 8 h p.i. total RNA. The nine events with reads in uninfected 4sU-RNA were only represented by very few reads, which can be attributed to noise or low levels of abnormal transcription termination and splicing in uninfected cells. These intergenic splicing events conclusively demonstrate that disruption of transcription termination results in large RNA molecules spanning two or more cellular genes.

### Disruption of transcription termination is host specific

**Figure 9:**
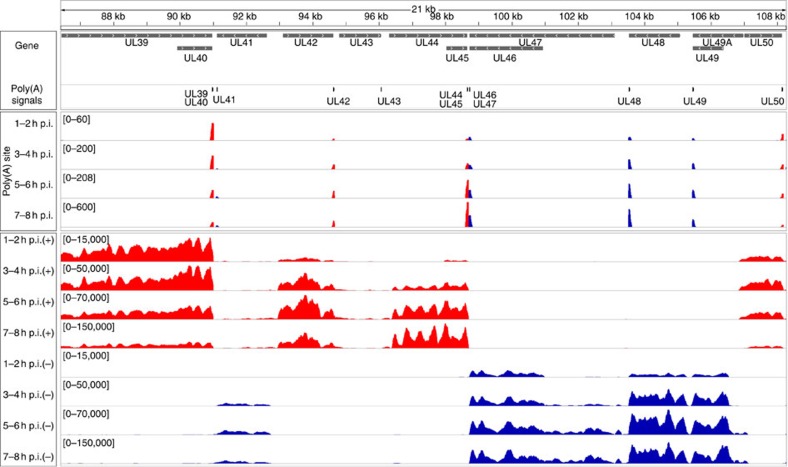
Disruption of transcription termination is host specific. Transcription and poly(A) site usage in a representative section of the HSV-1 genome. Top panel: annotation of viral genes and poly(A) signals is depicted in grey for 13 HSV-1 genes. Gene orientation is indicated by arrowheads. Middle panel: number of reads containing part of a poly(A) tail (red=forward strand; blue=reverse strand). Bottom panel: overall transcriptional activity in 4sU-RNA. Transcription of viral genes in 4sU-RNA is precisely limited to the annotated genes (HSV-1 strain 17; GenBank accession code: JN555585), which match the identified poly(A) sites. For each time point, the range of expression (*y* axis) is indicated in brackets (note: this increases throughout infection).

Transcription of viral genes followed the well-described cascade of immediate early, early and late gene expression (Supplementary Data 2). To analyse transcription termination in the viral genome, we identified reads containing part of a poly(A)-tail (see Methods). This allowed quantifying poly(A) site usage of all viral genes except the lowly expressed *RL1* gene (Supplementary Data 4; see Fig. 9 for the *UL39–50* gene segment). Viral poly(A) sites were almost exclusively (42/45) preceded by an AAUAAA poly(A) signal. Three additional, weakly expressed poly(A) sites were identified within the *UL24* and *UL44* gene, respectively, and on the positive strand between *UL55* and *LAT*. Regulated usage of the weak poly(A) site in the *UL24* locus has previously been reported^34^. To investigate changes in poly(A) site usage in the whole HSV-1 genome throughout infection, we correlated the gene expression upstream of each poly(A) site with the number of identified poly(A)-tailed reads (Supplementary Fig. 17). For 80% (38 of 48) of poly(A) sites, this correlation was >0.9. Only for 6% of sites, all of which had very low-read numbers, it fell below 0.8. This argues against regulated poly(A) site usage in HSV-1 infection and shows that disruption of transcription termination is host specific.

**Figure 10:**
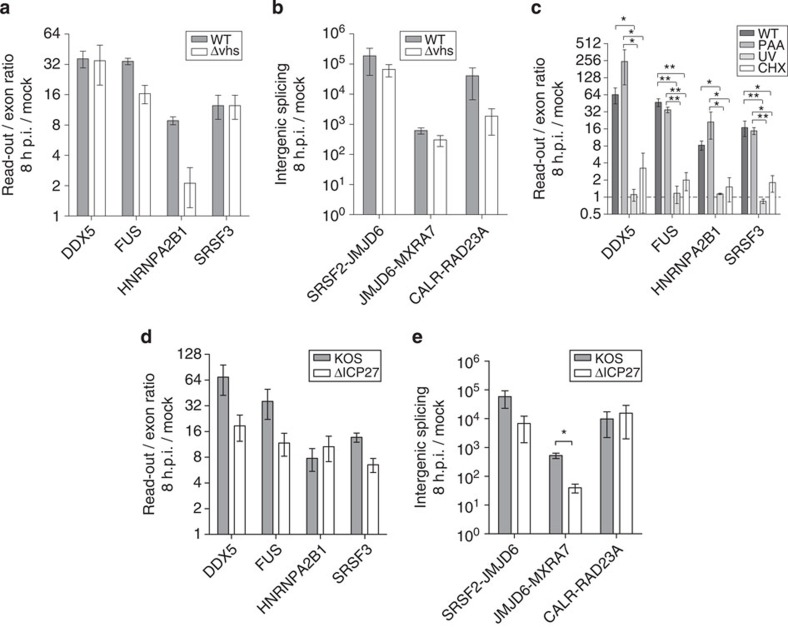
Disruption of transcription termination does not depend on vhs or ICP27. (**a**,**b**) *Vhs* is not required for disruption of transcription termination. Read-out was determined by qRT–PCR on total RNA following infection with wild-type HSV-1 or a *vhs*-null mutant (Δvhs). qRT–PCR was performed probing for transcription of the respective genes and downstream sequences (**a**) or intergenic splicing events (**b**) using exon–exon junction spanning primers. (**c**) Ultraviolet inactivation as well as CHX treatment abolished read-out, while PAA treatment had no effect. (**d**) HSV-1 KOS strain and its *ICP27*-null mutant (ΔICP27) both disrupt transcription termination. (**e**) Intergenic splicing was reduced >10-fold for two of three splicing events in ΔICP27 infection. (**a**–**e**) Bars show the average of three independent experiments (±s.e.m.). For pairwise comparisons (**a**,**b**,**d**,**e**) paired Student’s *t*-test was used. For multiple comparisons (**c**), one-way analysis of variance was used, followed by Tukey’s *post hoc* test. All significant *P* values (*<0.01, **P<0.001) are shown.

To investigate whether *vhs* was involved in disruption of transcription termination, we infected HFF with wild-type HSV-1 or a *vhs*-null mutant^35^ (Δvhs). We then employed qRT–PCR to probe for both read-out and intergenic splicing at 8 h p.i. in total RNA. Both were equally prevalent in wild-type and Δvhs HSV-1 infection (Fig. 10a,b). The two- to fourfold lower read-out/exon ratios observed for two of the four genes (*FUS* and *HNRNPA2B1*) in Δvhs infection resulted from higher total RNA levels compared with wild-type HSV-1 infection (Supplementary Fig. 3) reflecting the lack of *vhs* activity rather than reduced read-out^36^. Therefore, *vhs* is not required for the disruption of transcription termination. Furthermore, enhanced transcriptional activity downstream of poly(A) sites does not result from a relative increase in the background level of aberrant transcription termination due to *vhs*-mediated degradation of ‘normal’ transcripts.

We next studied read-out following infection with ultraviolet-inactivated virus as well as infection in presence of cycloheximide (CHX) or phosphonoacetic acid (PAA). CHX inhibits protein synthesis, whereas PAA only prevents viral late gene expression. While both ultraviolet inactivation and CHX treatment completely abolished read-out at 8 h p.i. in total RNA, PAA treatment had no effect (Fig. 10c). Thus, expression of viral true late genes is not required for HSV-1-induced read-out. Furthermore, disruption of transcription termination is not mediated by tegument proteins from invading virions, but requires *de novo* viral gene expression.

Finally, we employed an *ICP27*-null mutant^7^ to test whether the expression of *ICP27* was required. Both wild-type HSV-1 KOS strain and its *ICP27*-null mutant (ΔICP27) triggered read-out at 8 h p.i. in total RNA comparable to HSV-1 strain 17 (Fig. 10d). The two- to fourfold lower levels of read-out/exon ratios observed for three of four genes in ΔICP27 infection mainly resulted from higher transcript levels due to reduced vhs activity^36^. Intergenic splicing, however, which was not detectable by qRT–PCR in uninfected cells, was reduced >10-fold in infected cells for two of the three intergenic splicing events (Fig. 10e). We conclude that *ICP27* is not required for disrupting transcription termination but may contribute to aberrant splicing in HSV-1 infection.

## Discussion

We performed a comprehensive analysis of the temporal changes in host and viral transcriptional activity and RNA processing as well as their impact on translation during lytic HSV-1 infection. Strikingly, we found that HSV-1 induces the widespread disruption of transcription termination of cellular genes. Of the ∼10,000 cellular genes under study, 53% showed >35% read-out. Although changes in cellular gene expression were dominated by the downregulation of ∼75% of cellular genes by >2-fold and ∼6% by 10-fold in total RNA at 8 h p.i., disrupted transcription termination resulted in a stronger decline in translation rates of cellular genes with >35% read-out than for genes without read-out. This may be either due to poor nuclear export or impaired translation of the long read-through transcripts and suggests that disruption of transcription termination contributes to HSV-1 host shut-off.

Read-out results in massive intergenic transcription downstream of poly(A) sites as well as read-in transcription into downstream genes and antisense transcription to genes on the opposing strand. Interestingly, a previous report indicated a proteasome-mediated degradation of the elongating form of RNAP II late in HSV-1 infection^16^. It is tempting to speculate that disruption of transcription termination, extensive intergenic/antisense transcription and RNAP II stalling contributes to degradation of RNAP II and loss of transcriptional activity late in infection. While disruption of host transcription termination has not been reported before for any pathogen, knockdown of the non-coding RNA *7SK* was recently shown to have this effect in embryonic stem cells^37^. In our study, however, *7SK* was one of the few cellular genes induced (∼2-fold) in both total and 4sU-RNA that showed neither read-in nor read-out. Therefore, disruption of transcription termination in HSV-1 infection does not result from a loss of *7SK*.

Analysis of cellular poly(A) signals relative to the extent of transcription read-out indicated that genes with non-canonical and likely weaker poly(A) signals were more strongly affected by disrupted transcription termination. Twenty-five years ago, McLauchlan *et al*.^38^ reported the exact opposite for two HSV-1 genes. They reported that the virus induces an RNA processing factor which enhances the usage of weak poly(A) sites in the viral genome late in infection. Our data allowed us to comprehensively study poly(A) site usage in the viral genome on a global scale at defined times of infection. This confirmed the existence of the alternative poly(A) sites previously reported^34^,^38^,^39^. However, they were weakly utilized and no evidence of differential poly(A) site usage was found. Interestingly, McGregor *et al*.^40^ showed that HSV-1 infection resulted in an increased binding of nuclear extract proteins to *in vitro* transcribed pre-mRNAs containing HSV-1 poly(A) sites of all temporal classes in an *ICP27*-dependent manner. These findings may explain why disruption of transcription termination was specific to cellular genes and viral genes were not affected. Although *ICP27* was not required for disruption of transcription termination, it may thus contribute to the maintenance of efficient termination of viral transcripts. However, it is important to note that the expression of ICP27 is required for full late gene transcription^16^,^41^. Therefore, other viral genes may be involved. Alternatively, HSV-1 poly(A) sites may simply utilize the same cellular mechanisms which maintain functional transcription termination of some cellular genes (for example, *GAPDH* and *β-actin*) throughout HSV-1 infection.

A second surprising observation of this study was that hundreds of cellular genes appeared to be transcriptionally but not translationally induced during infection. We show that this is caused by disrupted transcription termination of an upstream gene resulting in extensive read-in into the seemingly induced genes. Furthermore, novel intergenic splicing events occurred between exons of genes connected by read-out/read-in demonstrating that read-out indeed results in large transcripts spanning multiple cellular genes. Thus, our approach allows distinguishing a bona fide increase in transcriptional activity from seeming induction by read-in. For example, our data confirm previous observations of HSV-1-induced transcription and translation of the haemoglobin alpha 2 gene (*HBA2*), which is normally expressed only in cells of erythroid lineages (see Supplementary Data 1)^42^,^43^,^44^.

Our third key observation concerns the inhibition of splicing by HSV-1, previously reported to be mediated by *ICP27* (ref. 7). We are the first to investigate this in living cells at whole-transcriptome level. Analysing newly transcribed rather than total RNA, we studied processing of RNA molecules transcribed at defined times of infection and independent of pre-existing RNA. While we did observe aberrant splicing events late in infection, our data do not support a generalized inhibition of splicing in lytic HSV-1 infection. Instead, increasing intron levels at late stages of infection are explained by insufficient splicing of transcripts resulting from read-in. Genes not affected by read-in continue to be efficiently spliced throughout infection. Several studies showed binding and rearrangement of splicing factors by ICP27 (refs 8, 10, 11, 12). However, the latter was not sufficient to inhibit splicing^11^. Inhibition of splicing was directly demonstrated using nuclear extracts and *in vitro* synthesized pre-mRNAs^7^,^8^,^9^. However, this assay is not carried out in living cells, only tests for post-transcriptional splicing and ignores the co-transcriptional nature of RNA processing^45^. We recently found that >65% of introns in human B cells are already spliced and degraded in 4sU-RNA prepared after only 5 min of 4sU exposure^21^. Additional evidence for splicing inhibition by *ICP27* was provided by transfection experiments with a plasmid containing an intron in the 5′ non-coding region of the *CAT* gene^11^,^46^,^47^. This showed decreased levels of spliced *CAT* mRNA in HSV-1 infection; however, unspliced *CAT* pre-mRNA was not detected. In summary, 4sU-seq demonstrated that rapid co-transcriptional splicing of cellular genes is not notably inhibited during lytic HSV-1 infection. In addition, all four intron-containing HSV-1 genes (*ICP0*, *UL15*, *ICP22*, *ICP47*) were found to be spliced throughout infection.

Interestingly, we observed a significantly greater extent of impaired splicing in lincRNAs genes following read-in than of protein-coding genes. This may simply reflect poor annotation of lincRNAs and their introns or suggest differences in lincRNA splicing compared to splicing of protein-coding genes (for example, post- versus co-transcriptional). Furthermore, although HSV-1 infection did not inhibit co-transcriptional splicing, we found evidence for aberrant splicing during HSV-1 infection and *ICP27* appeared to be involved at least to some degree. Thus, our study suggests an alternative interpretation for the observed *ICP27*–spliceosome interactions. Indeed, a recent study of HSV-2 *ICP27* found that it promoted alternative splicing in the promyelocytic leukaemia (*PML*) gene, resulting in intron retention of only one intron, while removal of other introns was not inhibited^48^.

Finally, we would like to note that our approach is not only applicable to any virus or pathogen *in vitro* but could also be extended to *in vivo* applications^49^,^50^,^51^,^52^. Owing to its outstanding temporal resolution, it is particularly well suited for pathogens with short replication cycles and is useful to gain mechanistic insights into their interference with and regulation of RNA synthesis, processing, export and translation.

## Methods

### Viruses and cells

This study was performed using HSV-1 strain 17 and its *vhs*-inactivated mutant (Δvhs)^35^. In addition, read-out and intergenic splicing was assessed by qRT–PCR for HSV-1 KOS strain and its *ICP27*-null mutant (ΔICP27)^53^. Both viruses were kind gifts from Professor Rozanne Sandri-Goldin (University of California). Strain 17 virus stocks were produced in baby hamster kidney (BHK) cells (ATCC) grown in complete Dulbecco’s modified Eagle’s medium (DMEM) containing 10% fetal bovine serum (FBS), tryptose phosphate broth, 2 mM L-glutamine, penicillin (100 U ml^−1^) and streptomycin (100 μg ml^−1^) by infecting them at a multiplicity of infection of 0.01. Two days post infection, the medium was collected and placed on ice while the cells were lysed by freeze-thawing. Cell debris was removed by centrifugation for 10 min at 10,000*g*. The supernatant was added to the cell culture medium and virus was pelleted at 33,600*g*, 4 °C for 2 h. The pellet was resuspended, loaded onto a 5–15% ficoll gradient and spun at 24,600*g*, 4 °C for 2 h. The band containing virus particles was collected, diluted with PBS to 35 ml and spun at 38,050*g*, 4 °C for 2 h to pellet the virus. The pellet was resuspended in 1–2 ml DMEM, aliquoted and frozen at –80 °C.

HFFs were purchased from ECACC and cultured in DMEM with 10% FBS Mycoplex and 1% penicillin/streptomycin. The cells were expanded to passage 9, harvested and stored in liquid nitrogen. On thawing, the cells were grown to passage 13. For infection, cells at passage 13 were seeded at 3.5 × 10^5^ cm^–1^ onto 15-cm dishes for RNA extraction and a 12-well plate for immunecytofluorescence analysis. The following day, the cell medium was collected, split into two parts and virus was added to one half to obtain the multiplicity of infection of 10. The virus-containing medium was added to cells in 15-cm dishes and 12-well plates at equal volume-to-surface ratio. After 10 min, the cells were washed twice with PBS and the previously saved virus-free medium was re-applied. This time point was considered as *t*=0 h p.i. Reapplying the conditioned media ensured that we were not measuring the cell response to receiving fresh medium. The same infection conditions were also used for virus titrations on HFF.

To assure that all the cells were infected in these conditions, we analysed the expression of the ICP4 protein by immunecytofluorescence in parallel with the 4sU-tagging and ribosome profiling experiments. At 4 h p.i., the cells were fixed with 4% paraformaldehyde in PBS for 10 min and blocked with 10% FBS in PBS for 1 h. Anti-ICP4 antibody (1:2,000, Abcam) diluted in 1% FBS/0.1% saponin/PBS was added to the cells for 1 h, followed by 1 h incubation with FITC-conjugated secondary antibody (1:500, Abcam) and counterstaining with 4′,6-diamidino-2-phenylindole (Sigma) for 10 min. In all experiments sent for sequencing, >95% of cells stained positive for ICP4.

To block protein synthesis or viral DNA replication, CHX (final concentration 50 μg ml^−1^) or PAA (300 μg ml^−1^), respectively, were added to cultured cells 15 min before infection.

### Isolation of RNA fractions

4sU was added to the cell culture medium for 60 min at −1, 0, 1, 2, 3, 4, 5, 6, or 7 h p.i. (2 × 15-cm dishes per condition) to a final concentration of 500 μM. Subsequently, the medium was aspirated and the cells were lysed with Trizol (Invitrogen). Total RNA and newly transcribed RNA fractions were isolated from the cells as described previously^19^,^24^.

### qRT–PCR analysis

Total or newly transcribed RNA (300 to 800 ng) were treated with DNase I (Fermentas) and reverse transcribed with SuperScript VILO Mastermix (Invitrogen) as per the manufacturers’ instructions. Complementary DNA (cDNA) levels were analysed by qPCR using primers at 1 μM each, with carboxy-X-rhodamine (Promega) as passive reference dye, in 10 μl total volume. Primers and probes were designed using http://www.universalprobelibrary.com/. For PCR analysis, the samples were denatured at 95 °C for 3 min, followed by 40 cycles of 95 °C (3 s) and 60 °C (30 s). For two PCR assays (intergenic splicing events: *CALR*-*RAD23A* and *JMJD6-MXRA*) we used incorporation dye-based GoTaq qPCR MasterMix (Promega). Here a melting curve step was added to confirm the amplification of a single PCR product. For all other assays, we used TaqMan-based GoTaq Probe qPCR Master Mix (Promega). Specific Universal ProbeLibrary TaqMan probes (Roche) were added to a final concentration of 0.1 μM. Viral gene expression was normalized to RNA18S5. Read-out levels were normalized to the level of exonic sequence of the upstream gene. As intergenic splicing was not detectable in uninfected cells, C_T_ values for uninfected cells were arbitrarily set to 37 (∼1 copy). Student’s *t*-tests and one-way analysis of variance followed by Tukey’s multiple comparison test were performed on ΔC_T_ values. Relative differences were determined using the ΔΔC_T_ approach. A list of all primers and probes is included in Supplementary Data 5a.

### RNA-seq

For both total and 4sU-RNA samples, library preparation for sequencing was performed using the stranded TruSeq RNA-seq protocol (Illumina, San Diego, USA) with 800 ng RNA as input for each sample. For the total RNA libraries, both cytoplasmic and mitochondrial rRNA species were depleted using the Ribo Zero Gold kit (Epicentre, WI, USA). No rRNA depletion was performed for 4sU-RNA samples as rRNA only contributes about 40–50% of reads in 4sU-RNA samples. In the PCR step, to enrich for fragment-ligated adaptors, only 13 cycles were used, which minimized PCR bias. Barcoded libraries were subjected to sequencing by synthesis at 2 × 101 nt on a HiSeq 2500 (Illumina). Quality monitoring and output settings were handled according to standards described by ‘t Hoen *et al*.^54^.

### Ribosome profiling

Ribosome profiling was performed as described before^55^ with the following modifications (steps in brackets refer to the original protocol). Cells were infected in 15-cm dishes (one dish per condition) and lysed using 800 μl lysis buffer with flash-freezing (step 2B). Lysate (900 μl) was treated with 22.5 μl RNase I (step 7). Overnight gel extraction was used at every stage of the procedure (step 25). For every precipitation of RNA or cDNA, 1 volume of isopropanol, 1 μl of GlycoBlue and sodium acetate (where required) was added and the solution was incubated at –80 °C for 30 min. After centrifugation at 20,000*g* for 30 min, the pellet was washed with 80% ethanol. For ligation (step 31) an alternative primer was used (Supplementary Data 5b). The ligation mixture (step 32) was incubated at 16 °C overnight. The main deviation from the original protocol was barcoding the samples at the stage of reverse transcription (steps 37–38), and not PCR amplification, which reduces the risk of cross-contamination between samples (for details, see Supplementary Data 5b). The whole 20 μl of the circularization reaction (step 47) was used for rRNA depletion. Additional biotinylated oligos (Supplementary Data 5b) were included to more efficiently remove rRNA from the samples. Streptavidin clean-up was performed twice, instead of only once, to ensure that all biotinylated oligos were removed before PCR amplification. We found this to substantially increase the specificity of the final library amplification resulting in cleaner libraries. After rRNA depletion, the following linearization step was added to the procedure. Circular cDNA pellet was resuspended in 30 μl linearization mix containing 1 μl 10 μM linearization primer, 3 μl FastDigest Buffer (Fermentas) and 26 μl water. The solution was heated to 95 °C for 2 min and cooled at 1 °C per minute to 25 °C, at which point 2 μl of FastDigest BamHI (Fermentas) was added. After 30 min incubation at 37 °C, the cDNA was precipitated with isopropanol/ethanol, as described above. The pellet was resuspended in 12 μl water and 0.2 μl was used for optimization PCR with Solexa primers (P5and P3) and AccuPrime SuperMix I (Invitrogen). Reactions were carried out by denaturing at 94 °C for 2 min, followed by test PCRs running for 7, 10, 13 or 16 cycles of: 94 °C for 15 s, 65 °C for 15 s and 68 °C for 30 s, with final extension at 68 °C for 3 min. Products were separated on polyacrylamide gel (steps 59, 60). On the basis of this result, the optimal number of cycles was chosen to amplify the bulk of the linearized product, which was subsequently purified from the gel and sent for sequencing at the Beijing Genomics Institute (BGI). All libraries were quality checked by Agilent Bioanalyzer and sequenced on a HiSeq2000 with 50 nt single-end reads following the manufacturer’s instructions.

### Mapping of RNA-seq data (total RNA and 4sU-RNA)

RNA-seq data were mapped in parallel against human and HSV-1 genomes as well as rRNA sequences using ContextMap^56^. Paired-end (2 × 101 nt) RNA-seq reads were mapped in parallel against (i) the human genome (GRCh37/hg19), (ii) human rRNA sequences that are not included in the human reference assembly and (iii) the HSV-1 genome (Human herpesvirus 1 strain 17, GenBank accession code: JN555585). For the two repeat regions in the HSV-1 genome, only one copy of each was retained. Thus, nucleotides 1–9,213 and 145,590–152,222 were excluded from the alignment. Parallel mapping was performed using ContextMap v2.3.0 (with BWA as integral short-read aligner^33^) as described previously^56^.

In brief, the ContextMap approach consists of five steps:

#### Step 1: identification of initial alignments

In this step, all reads are first aligned against all references sequences using a short-read alignment programme, in this case BWA^33^. This allows identifying continuous alignments of (part of) the read against the used reference sequences.

After obtaining initial alignments, all alignments with a maximum number of mismatches (four for this study) are then categorized as either

- Full alignments if they can be aligned continuously and end-to-end to the genome
- Candidate split alignments if either the start or the end of the read cannot be aligned and the unaligned read parts exceeds a certain length threshold
- Partial alignments if the unaligned read parts are shorter than this threshold
- Candidate multi-split alignments if both read start and end cannot be aligned. This allows determining reads crossing multiple exon–exon junctions. This option is only available, if a short-read aligner is used that determines local alignments, such as BWA.

Candidate split alignments represent an alignment of only a part of the read, that is, either the start or the end of the read. If the read contains an exon–exon junction, the rest of the read can only be aligned to the genome at some distance to this alignment. The gap in the alignment represents the spliced intron. State-of-the-art short-read alignment programs, such as BWA, do not allow identifying alignments with such large gaps, thus the following approach is used to identify the second part of the alignment. First, the alignment of part of the read is used to define a smaller window of the genome of at most two million nt. The short-read alignment programme is then again used to find an alignment of the previously unaligned part of the read in this window. The two alignments of the start and end of the read are then combined to a final split alignment.

Candidate multi-split alignments (that is, alignments crossing more than one exon–exon junction) are essentially treated as special cases of candidate split alignments (representing one exon–exon junction only) by fragmenting the read based on the local alignment identified for this read.

#### Step 2: identification of contexts

Alignments of reads to the same genomic region are clustered into contexts if they are at most a predefined genomic distance apart. For all following steps until the last one, contexts are treated independently of each other and several alignments of each read to either several contexts or several positions within the same context are evaluated. This allows parallel evaluation of different read sources, such as in this case the human and HSV-1 genome and rRNA sequences.

#### Step 3: alignment extension

In this step, additional split alignments are determined for full, partial and split alignments based on the splice junctions indicated by the split alignments determined previously. The purpose of this step is to identify all possible alignments for a read that do not exceed a predefined maximum mismatch criterion.

#### Step 4: within-context resolution of multiple alignments

The purpose of this step is to identify the best alignment for each read within each context based on all other read alignments in this context. For this purpose, the best splice sites among overlapping splice sites are determined first. If at least one of these splice site has a known splice signal (=last two nucleotides on both ends of the intron), all other overlapping splice sites without a known splice signal are discarded. Among the remaining overlapping splice sites, the three splice sites with the highest support by different read alignments are retained and all others (including the corresponding alignments) are discarded. Subsequently, a support score for each remaining read alignment is calculated as a weighted sum of maximum read coverages in predefined windows around the read alignment. Among multiple alignments for a read within the context, the one with the highest support score is then chosen.

#### Step 5: between-context resolution of multiple alignments

After step 4, each read is aligned to only one position in each context, but can still be aligned to more than one context. These multiple alignments are resolved in the same way as in step 4 by recalculating support scores and then choosing the alignment with the highest support score. At the end of this step, each read is aligned to at most one context in one reference sequence, that is, either rRNA sequences or the human or HSV-1 genome.

### Processing and mapping of ribosome profiling data

Ribosome profiling data (50 nt single-end reads) were processed using the following in-house pipeline:

#### Adapter trimming

The 3′ sequencing adapter sequence was removed from the 50 nt reads by computing a prefix–suffix sequence alignment between adapter and read sequences (match score: 1; mismatch score: −4; gap open penalty: −10; gap extend penalty: −2; leading gaps in read sequence and trailing gaps in adapter sequence not penalized). Reads were then trimmed to remove the suffixes with positive alignment score to the adapter sequence.

#### Barcode removal

Barcode sequences (first 9 nt of read=3 random bases followed by 4 bases of sample-specific barcode followed by 2 random bases) were removed from all reads and stored for later use.

#### Mapping

Bowtie 0.12.7 (ref. 57) was used to align all reads to the genome and rRNA sequences used for RNA-seq mapping. In addition, alignment was performed against human transcripts from Ensembl (v75), HSV-1 transcripts as well as novel junctions identified in the 4sU data by ContextMap. Owing to the small read size (∼29–30 nt after barcode and adapter removal), we used a short-read alignment programme (performing no splice site prediction) to align against genome and transcript sequences, as prediction of splice sites using RNA-seq mapping programs is unreliable for such short reads. Furthermore, we used Bowtie instead of BWA as it is more precise for short reads^58^. All optimal Bowtie alignments with at most two mismatches were used. Only novel junctions with at least five distinct reads for at least one time point in 4sU-RNA were used. To obtain junction sequences for the alignment, 30 nt on both sides of the junction were extracted from the human genome. For transcriptome alignments, genomic positions were calculated and integrated with genome alignments. Only reads aligning to at most three distinct genome positions were kept for further analyses.

#### Assignment to time points and mismatch correction

To identify PCR duplicates, the reverse transcription primers used for library preparation contained five random bases flanking the sample-specific barcodes. Thus, PCR duplicates of the same RNA fragment match at these five positions, whereas distinct RNA fragments differ in these random barcode sequences. To calculate the number of distinct RNA fragments before PCR for each alignment position (denoted as footprint count), we first identified PCR duplicates as described below. Each set of PCR duplicates was then counted only once for the footprint count.

#### Identification of PCR duplicates at each alignment position

First, the most frequently observed sequence (composed of random barcode and ribosome footprint sequence) was determined. Second, this sequence and all sequences with exactly one mismatch (to account for sequencing errors) were removed. This procedure was repeated until no sequences remained. The number of iterations was used as the footprint count. This procedure was repeated separately for each sample-specific barcode. Barcodes not matching any sample-specific sequence were discarded.

### Identification of open reading frames by ribosome profiling

Using the combined alignments, cellular and viral open reading frames (ORFs) were identified as follows. Each chromosome and strand was scanned for clusters of overlapping ribosome footprint sequences. For each cluster, a weighted directed acyclic graph (DAG) was constructed. The vertices of the DAG represented the footprint sequences weighted by their total footprint count. Two sequences were connected by a 5′ to 3′ directed edge, if they were in-frame (the start position difference was divisible by 3 when considering introns), not too far apart (at most 50 triplets when considering introns) and there was no other read or in-frame stop codon in between. The path with the highest weight in this DAG was then determined iteratively and removed. Only paths passing the following filters were considered ORFs: on average, at least 0.1 fragments per triplet, at least 20 footprints in total and 10 distinct footprints, a stop codon at most 10 triplets away from the end of the path and the total number of footprints in the path exceeded 10% of the total number of footprints in the complete DAG.

### Quantification of gene expression

Gene and transcript annotation were obtained from Ensembl (v75). Expression levels of genes, exons and introns were estimated using the standard RPKM measure (=number of reads per kilobase of gene, exon or intron per million mapped reads)^59^. Number of reads mapping to a gene were determined as the total number of exon and exon–exon junction reads for this gene.

### Normalization of RPKM values

Total numbers of mapped reads for normalization were determined as follows:

#### RNA-seq data

Our data showed that RNA transcription (reflected by 4sU incorporation rates) remained rather stable during the first 8 h of HSV-1 infection (see Supplementary Fig. 1). Since rRNA transcription constitutes the bulk of transcription, this indicates that rRNA transcription remains stable. We exploited the fact that 4sU-RNA was not rRNA depleted to determine whether transcription of viral genes replaced transcription of host genes or occurred in addition to host transcription. Relative to rRNA read numbers, number of reads mapping to the host genome remained approximately stable while the number of reads mapping to either host or viral genome increased. This indicates that total RNAP II transcription increased throughout the first few hours of infection due to the advent of viral gene expression. Thus, to account for this additional transcription, normalization of 4sU and total RNA was performed against the number of reads mapping to the host genome. Only genes with an RPKM⩾1 for at least one time point were included in the analysis.

#### Ribosome profiling data

For ribosome profiling data, no control was available to evaluate whether the extent of translation changed throughout infection. Translational upregulation correlated better to transcriptional activity across time points when we normalized the ribosome data to the total number of reads mapping to either host and virus genome. Therefore, we used this normalization for our analysis. It is, however, important to note that the analysis presented in the article on translational activity and its correlation to transcriptional activity does not depend on the normalization used for ribosome profiling. Calculation of correlation between samples is scale-invariant, that is, multiplication by a constant for both samples does not change the value of the correlation coefficient. For calculating the fraction of transcriptionally upregulated genes that are translated, we only evaluated whether a translated ORF was observed at all (i.e., RPKM>0) in 7–8 h p.i., not the level of translation.

### Identification of up- and downregulated genes

Regulated genes were identified based on two criteria:

1. A fold-change ⩾2 between uninfected cells and 7–8 h p.i. for 4sU-RNA and 8 h p.i. for total RNA and ribosome profiling, respectively.
2. A consistent increase (for upregulated genes) or decrease (for downregulated genes) across the whole duration of lytic HSV-1 infection. A consistent increase or decrease was identified by a rank correlation coefficient ⩾0.3 or ≤−0.3, respectively, between the duration of infection and the gene expression level.

### Random sampling of non-induced genes

To confirm that the low number of translated ORFs at 8 h p.i. for transcriptionally induced genes was not simply due to low total RNA levels, numbers of translated ORFs were also evaluated for randomly sampled gene sets containing the same number of non-induced genes with approximately the same RPKM (±0.1) as the induced genes in total RNA at 8 h p.i.

Sampling of one random gene set *S* was performed by iteratively sampling a random non-induced gene with similar expression for each transcriptionally induced gene *G*. This was done by first determining all non-induced genes that had the same RPKM±0.1 as *G* in total RNA at 8 h p.i. and were not yet included in *S*. From these genes, a random gene was then sampled and added to *S*.

Sampling was repeated independently 1,000 times and the percentage of translated ORFs at 8 h p.i. was determined for each sample. Distributions of these percentages were visualized using boxplots. *P* values were calculated by determining the fraction of random samples having equal or lower numbers of translated ORFs than the induced genes. Since none of the random samples had such low numbers, this results in *P* values<1/1,000=0.001.

### Quantification of read-out and read-in

To evaluate the degree of read-out or read-in, we determined the number of reads mapping in a window downstream of the 3′-end or upstream of the 5′-end of each gene in 7–8 h p.i. 4sU-RNA. Window size was determined as 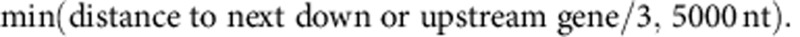


If the distance to the next downstream or upstream gene was <300 nt, that is, for a window size <100 nt, the gene was not included in the analysis. RPKM values for this window at 7–8 h p.i. were calculated as for gene regions and divided by the gene RPKM at 7–8 h p.i. to quantify the extent of read-out or read-in. If this ratio was >5%, a positive rank correlation (⩾0.3) between the duration of virus infection and the RPKM downstream or upstream of the gene was also required to indicate read-out or read-in. If the rank correlation was <0.3, that is, there was no increase in transcriptional activity downstream or upstream of the gene during virus infection, the extent of read-out or read-in was classified as ‘unclear’.

### Functional enrichment analysis

Functional enrichment within genes with >75% read-out was calculated using the DAVID webserver^60^ with default parameters and against default databases (OMIM, GO, BBID, Biocarta, KEGG, InterPro, PIR, SMART). As background, we used the 9,690 genes for which read-out and read-in were determined. Multiple testing correction was performed with the method by Benjamini–Hochberg^61^ and a *P* value threshold of 0.05 was used.

### Quantification of the fraction of unspliced transcripts

To quantify the fraction of transcripts that are not completely spliced, we calculated intron/gene ratios as the ratio of intron RPKM to gene RPKM in 4sU-RNA. For the analysis of intron/gene ratios of highly expressed genes, we selected genes with an RPKM ⩾10 in uninfected 4sU-RNA as well as introns with an intron RPKM ⩾1 in all 4sU-RNA samples (2,013 cellular genes). This was done to have sufficiently large read numbers for a reliable statistical analysis.

### Identification of induced splice junctions

Induced splice junctions were identified by comparing the following two ratios for any splice junction between two annotated exons:




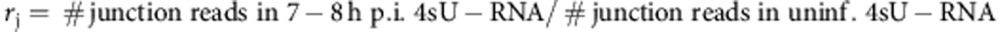




and




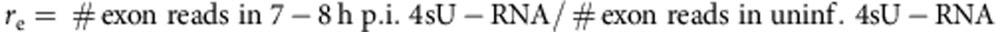




Junction reads were defined as all exon–exon junction reads for this particular splice junction. Exon reads were all (unspliced) reads completely mapping to exonic regions of the corresponding gene. A pseudocount of 1 was used in all cases. We evaluated only junctions for which at least one read in each replicate and at least five reads in total were observed either in the uninfected sample or at 7–8 h p.i. A particular splice junction was classified as induced if the odds ratio *r*_j_/*r*_e_ was at least 10 and statistically significant (Fisher’s exact test corrected for multiple testing ≤0.05). Multiple testing correction of *P* values was performed with the method of Benjamini and Hochberg^61^. Induced junctions were classified as novel if they were not observed in any transcript annotated in Ensembl, intergenic if they connected annotated exons of two different genes, and nonsense-mediated decay (NMD) associated if all transcripts containing that junction were annotated with NMD in Ensembl.

### Identification of high-confidence intergenic splicing events

To exclude that the induced intergenic splicing events were an artefact of the RNA-seq mapping approach, we pursued the following approach:

1. We generated a database of possible intergenic splicing events between all pairs of genes that were ≤100 kb apart (database A). We did this by generating a list of 160 nt junction sequences containing 80 nt of both flanking exons. Here, all possible pairs of exons for each pair of genes were used. Thus, any 101 nt sequencing read mapping to one of these *in silico* generated splice junctions would have at least 21 nt mapping to either exon. Since gene annotations differed between the Ensembl annotation and the UCSC gene annotation, we furthermore removed all junction sequences from the database that fell within a gene in the UCSC gene annotation.
2. We realigned all sequencing reads in the uninfected and 7–8 h p.i. 4sU-RNA samples using BWA. Parameters were set such that only alignments of the complete 101 nt read were determined. Only alignments with at most five mismatches were retained. For reads that had already been aligned in the original mapping, only alignments were retained that had less mismatches than the original mapping or were aligned to the same genomic location as in the original mapping. In addition, an intergenic alignment of a read was only retained if the second read of the pair was mapped to either of the two genes involved in the intergenic junction in the original mapping.
3. We again retained only intergenic junction events that were found with at least one read in each replicate and at least five reads in total for either the uninfected or 7–8 h p.i. samples.

As a control, we performed the same approach for a database that contained the same splice junctions as above but with the order of the corresponding exons exchanged (database B). This means that the 3′-end of the exon of the downstream gene was connected to the 5′-end of the exon of the upstream gene. Such splicing events cannot occur on a bicistronic transcript arising from read-out/read-in. Thus, they could only arise either from trans-splicing between two mRNAs or mapping errors due to repetitive regions in the genome. In the control database B, we identified 18 ‘intergenic’ junctions, which corresponded to only six genomic regions. Five of these regions accounted for 64% (16 of 25) of intergenic junctions from database A for which we found reads in uninfected cells. Thus, these are likely false positive results found due to repetitive sequences in these genomic regions. As a last filtering step, we removed any intergenic junction from database A falling into these regions as false positives, resulting in the final set of 71 high-confidence intergenic splice junctions.

### Quantification of poly(A) site usage

To quantify poly(A) site usage, we used the following approach for mapping reads containing a poly(A) tail. First, unmapped reads were realigned to the genome using the short-read alignment programme BWA^33^, which allows the identification of so-called clipped read alignments in which only parts of the read are aligned. Since the poly(A) tails are not encoded in the genome, any read covering the poly(A) tail can only be aligned partially to the genome. Here only one read from each read pair was used, that is, the read from the start of the fragment containing the poly(A) tail. Sequencing quality for the second read, which would be starting with the poly(A) sequence, was generally very poor, likely due to technical problems of sequencing long repeats of the same nucleotide.

Second, for all clipped read alignments for which only the start, but not the end, of the read was aligned, we checked for the presence of a poly(A) sequence in the 10-nt downstream of the aligned read part. If the unaligned read part was shorter than 10 nt, the complete unaligned read part was investigated. If this was shorter than 5 nt, the read was discarded. If the 5–10 nt sequence contained at most one non-A nucleotide, the read was considered a poly(A) read.

The position of the poly(A) sites was determined as the end of the aligned region of the read. As the end of the alignment could vary by a few bases for the same poly(A) site, we clustered reads into one poly(A) site if the predicted poly(A) sites were at most 25 nt apart. Finally, only poly(A) sites were retained that were supported by at least three reads with distinct starting positions in the genome in at least three samples. This was done to ensure that they did indeed originate from distinct fragments and not PCR duplicates.

### Identification of poly(A) signal sequences

The current consensus sequence for mammalian poly(A) signals consist of^28^:

- an AAUAAA (or closely related) sequence 15–30 nt upstream of the cleavage site
- a U-rich sequence 0–20 nt upstream of the AAUAAA sequence
- a G/U- or U-rich sequence 0–20 nt downstream of the cleavage site

To investigate whether there was a correlation between read-out and the presence of different poly(A) signals, we first extracted 50 nt upstream of each gene’s 3’-end (sequence set A, 9,668 genes, genes with the last UTR<50 nt were excluded) and mined these for the occurrence of common motifs. Motif search was performed using the DREME (Discriminative DNA Motif Discovery)^62^ tool contained in the MEME motif search suite^63^. This identified the motif shown in Supplementary Fig. 18 corresponding to the two most frequent poly(A) signal sequences previously reported, namely AAUAAA and AUUAAA.

Motif occurrences within the last 50 nt of each gene were then identified with the FIMO (Find individual motif occurrences) tool of the MEME suite using a *P* value threshold of 0.01. This also identified occurrences of related sequences differing at one position. If more than one motif match was identified for a gene, the one with the lowest *P* value was chosen. Frequency of different variants of this motif was then compared between genes with ≤5% read-in but different extent of read-out.

Motif search was also performed using DREME in the 50-nt downstream of the gene 3′-end (sequence set B) as well as in the 50-nt upstream of the A[A/U]UAAA motif match identified above (sequence set C, contains only 8,674 genes that had a A[A/U]UAAA motif match). In the first case, the best motif was U[C/G/A]U[U/G]U; however, this was found only 1.2 times more often in the true sequences compared to randomly shuffled ones (6,275/9,690 versus 5,159/9,690). In the second case, the best motif was UGUA[U/A/G], but again this was found only 1.6 times more often than in randomly shuffled sequences (3,179/8,674 versus 2,048/8,674). In comparison, the canonical A[A/U]UAAA motif was found 3.6 times more often in the true sequences than in the shuffled sequences (7,287/9,668 versus 1,892/9,668). The high frequency of the motif matches in randomly shuffled sequences for sequence sets B and C likely indicate that these are mostly found to relatively high G/U-content downstream of gene 3′-ends and upstream of the A[A/U]UAAA motif. This would be consistent with the fact that no proper motif has been previously described for these parts of the poly(A) signal.

Finally, we calculated position-specific nucleotide frequencies in a 200-nt window around the gene 3′-ends as in ref. 64 (see Supplementary Fig. 5). This clearly recovered the peak in A’s 20–30 nt before the gene 3′-ends corresponding to the A[A/U]AAA signal. In addition, genes with >35% read-out (but ≤5% read-in) had a slightly lower frequency of A’s in this region than genes with ≤5% read-out, consistent with the higher frequency of non-canonical signal sequences for these genes.

Furthermore, comparison for the regions before the A[A/U]AAA signal and after the gene 3′-ends showed some differences. In general, genes with >35% read-out tended to have more A and U’s than genes with ≤5% read-out and less C and G’s. However, the implications of these differences with regard to poly(A) signal strength are not quite clear as the influence of the sequences upstream of the A[A/U]UAAA motif and downstream of the cleavage site is not well understood.

## Additional information

**Accession codes:** All sequencing data have been deposited in the Gene Expression Omnibus (GEO) database with accession codes GSE59717 (4sU-tagging and total RNA) and GSE60040 (ribosome profiling).

**How to cite this article:** Rutkowski, A.J. *et al*. Widespread disruption of host transcription termination in HSV-1 infection. *Nat. Commun.* 6:7126 doi: 10.1038/ncomms8126 (2015).

## Supplementary Material

Supplementary Figures Supplementary Figures 1-18

Supplementary Data 1 Expression values of cellular genes in 4sU-RNA, total RNA and ribosome profiling (RP) as well as percentage of read-out and read-in.

Supplementary Data 2 Expression values of viral genes in 4sU-RNA, total RNA and ribosome profiling (RP).

Supplementary Data 3 Intergenic splicing events identified with high confidence.

Supplementary Data 4 Poly(A) sites identified in the HSV-1 genome.

Supplementary Data 5 PCR primers and probes (a), and list of DNA oligos used in ribosome profiling (b).
