# Supplementary material for: Widespread disruption of host transcription termination in HSV-1 infection
Source: Nat Commun. 2015 May 20;6:7126. doi: 10.1038/ncomms8126 (PMC4441252; doi:10.1038/ncomms8126)
Supplement: Supplementary Figures — 1-18 [file ncomms8126-s1.pdf]

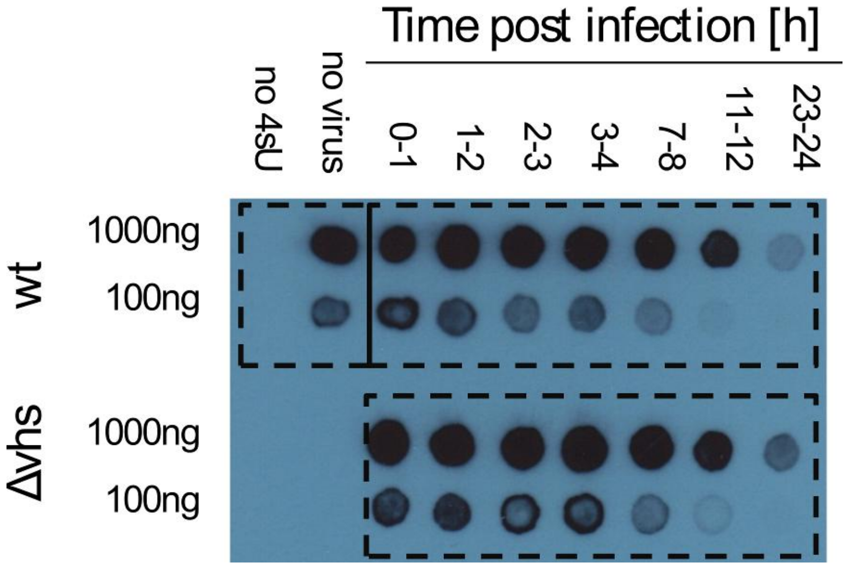

Supplementary Figure 1: 4sU incorporation throughout HSV-1 infection

HFF were infected with wild-type HSV-1 or its Δvhs mutant at an MOI of 10. 4sU-tagging was performed in one-hour intervals at the indicated times of infection. Total RNA was extracted and 4sU residues were thiol-specifically biotinylated. To evaluate transcriptional activity, 4sU incorporation in biotinylated total cellular RNA was determined by dot blot applying 1µg and 100ng biotinylated RNA as described[1]. Following a mild increase at 1-2h p.i., 4sU incorporation slowly declined with similar kinetics for both viruses. While 4sU incorporation decreased only by approximately 2-fold until 7-8h p.i., it dropped drastically by 23-24h p.i.

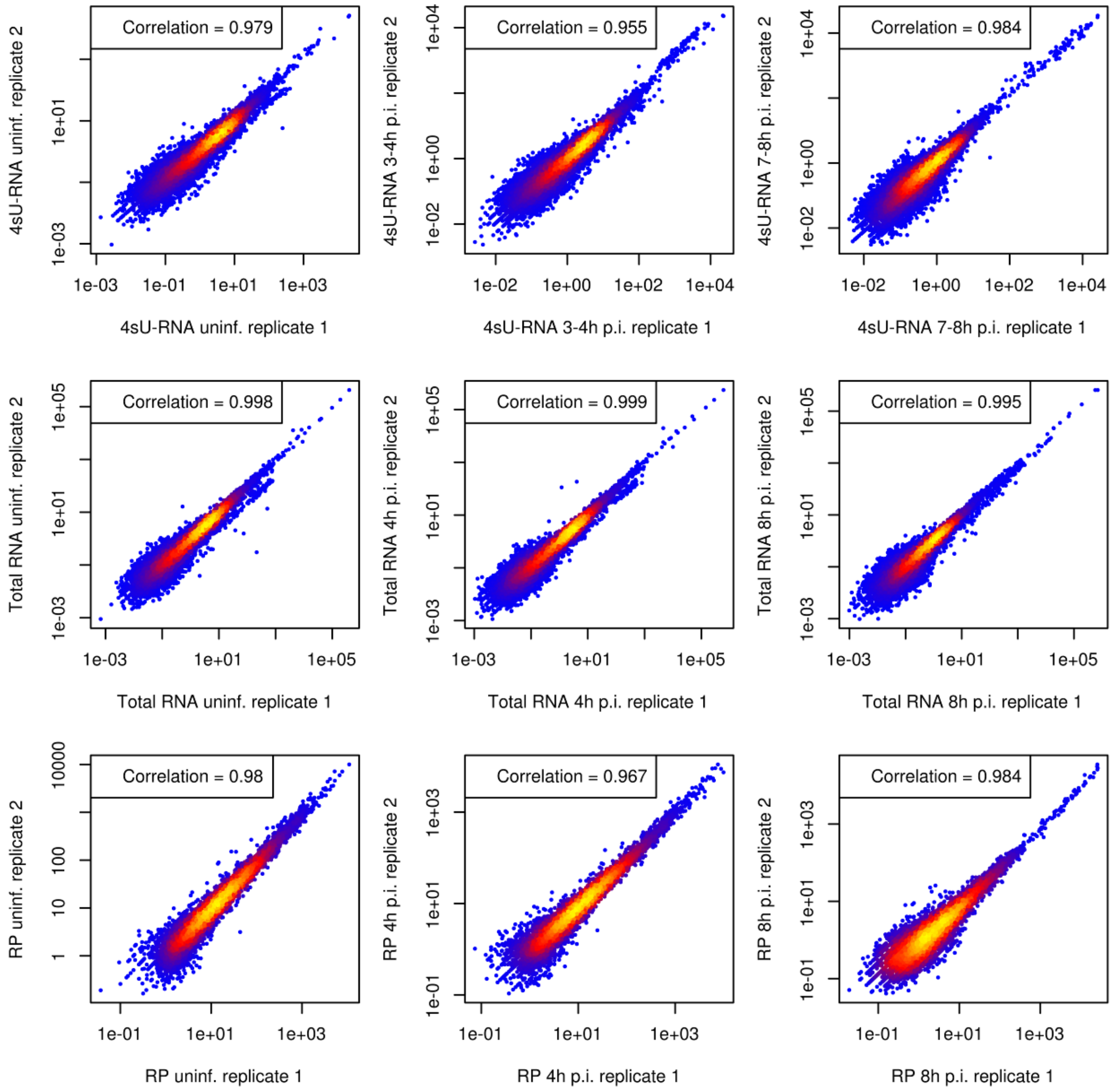

Supplementary Figure 2: 4sU-tagging and ribosome profiling experiments are highly reproducible

Comparison of RPKM (=reads per kilobase per million mapped reads) for human and viral genes between the two replicates shows high correlation for 4sU-RNA (top panels), total RNA (middle panels) and ribosome profiling (bottom panels). Three exemplary results each are shown for measurements in uninfected cells and 4 and 8h p.i. Density of points is colour-coded: yellow=high density, blue=low density.

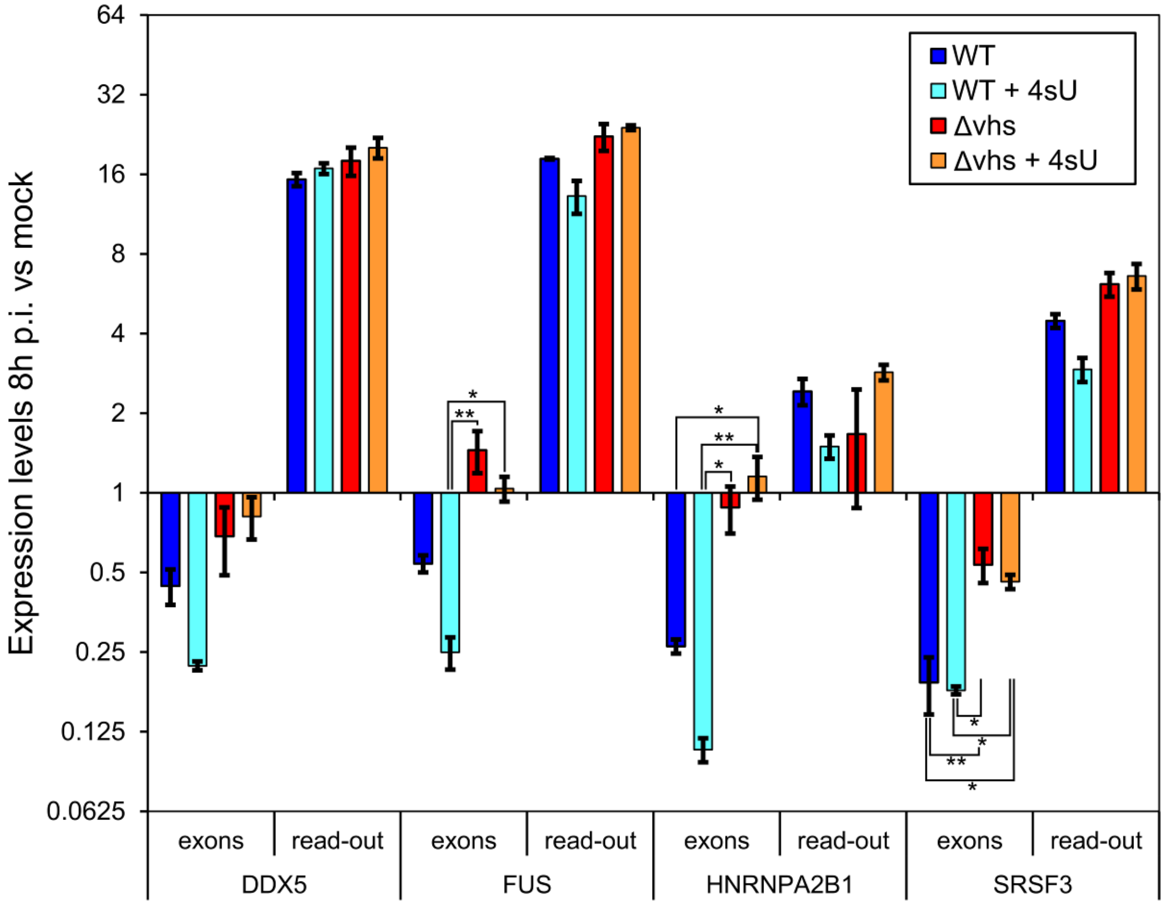

Supplementary Figure 3: Disruption of transcription termination is independent of 4sU exposure and does not require vhs

HFF were infected with wild-type HSV-1 or a *vhs*-null mutant (Δvhs). Total RNA was isolated at 8h p.i. either with or without adding 500µM 4sU 1h prior to RNA isolation. qRT-PCRs were performed using both exon-spanning PCRs (to quantify the gene's transcription level) and sequences located downstream of the respective gene's poly(A) site. Expression levels at 8h p.i. were normalised to expression levels in uninfected cells. Incubation with 4sU showed no effect on the disruption of transcription termination. Exon levels in Δvhs infection were 2- to 8-fold higher than in wild-type HSV-1 infection explaining the slightly reduced read-out/exon ratios in Δvhs infection for HNRNPA2B1 in Fig. 10a (one-way ANOVA analysis, followed by Tukey's post-hoc test: * $p<0.01$; ** $p<0.001$).

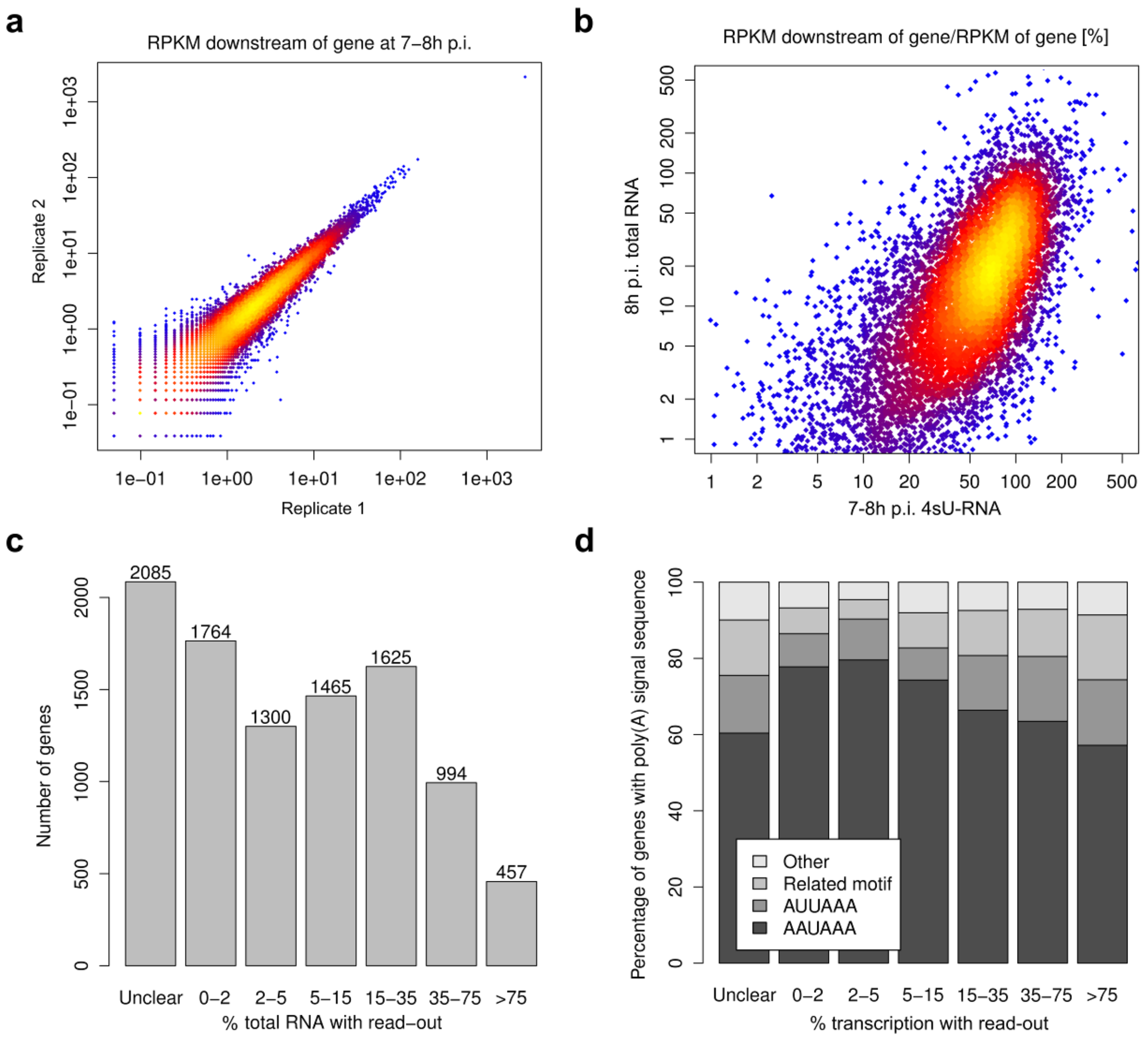

Supplementary Figure 4: Disruption of transcription termination is observed in total RNA and correlates with poly(A) signal sequences

(**a**) RPKM values downstream of gene 3'-ends are highly reproducible between replicates as shown for 7-8h p.i. 4sU-RNA. (**b**) Read-out is also observed in total RNA and is significantly correlated to the extent of read-out in 4sU-RNA (quantified as 100 x RPKM downstream of gene/gene RPKM at 8h p.i. for total RNA and at 7-8h p.i. for 4sU-RNA). (**a, b**) Density of points is colour-coded: yellow=high density, blue=low density. (**c**) Number of genes with different extent of read-out in total RNA (percentage of read-out in total RNA = 100 x RPKM downstream of gene/gene RPKM at 8h p.i.). The contribution of transcripts with read-out in 4sU-RNA is larger than in total RNA as the latter to a large degree represents transcripts produced prior to or during early stages of infection. In contrast, 4sU-RNA represents RNA transcribed at defined stages of infection only. As a consequence, fewer genes show a high extent of read-out in total RNA compared to 4sU-RNA. (**d**) The frequency of poly(A) signal sequences is correlated to the extent of read-out. Occurrence of signal sequences in the

50nt upstream of gene 3'-ends were determined as described in Supplementary Methods. The canonical AAUAAA motif is enriched within genes with low read-out, whereas the less common AUUAAA sequence and other motif hits differing at one position from the A[A/U]UAAA consensus ("related motif") are enriched within genes with high read-out. "Other" indicates that no match of the A[A/U]UAAA motif or a related sequence was found.

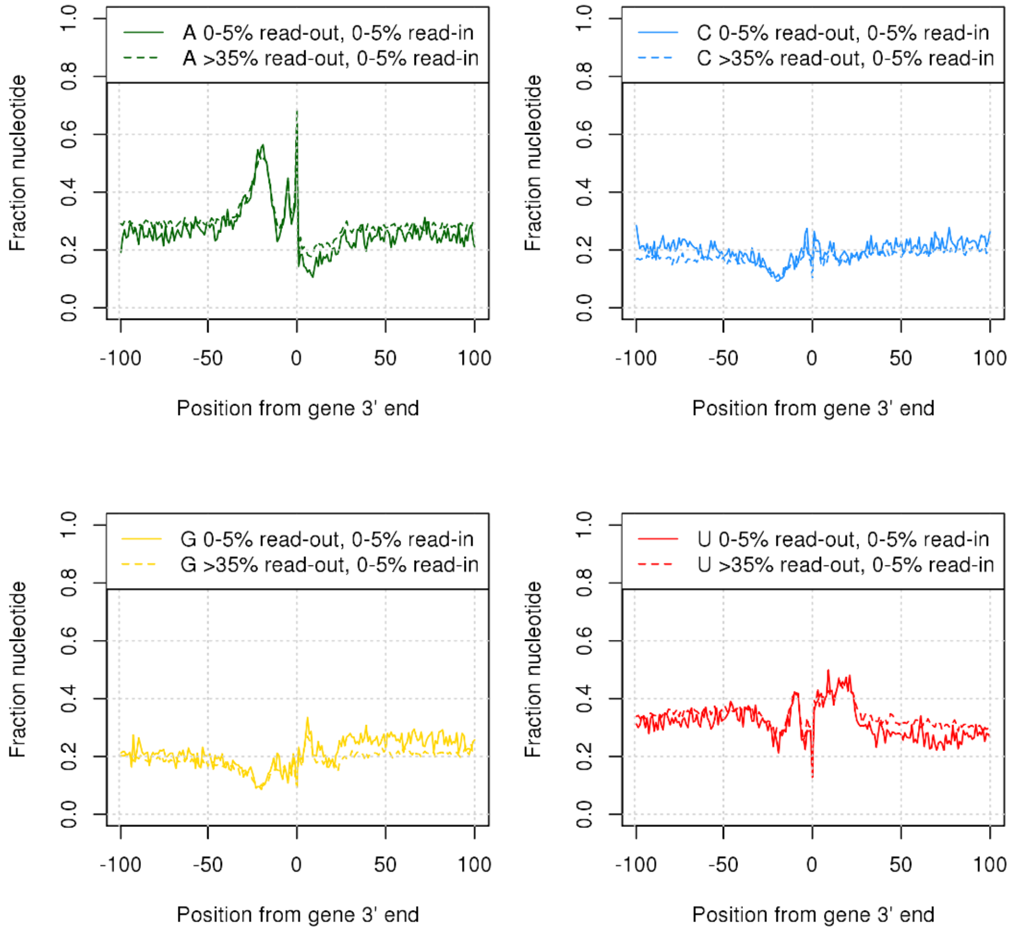

Supplementary Figure 5: Nucleotide frequencies around gene 3'-ends differ between genes with little or high read-out

Relative frequency of A, C, G, and U were calculated within 100nt around gene 3'-ends and compared between genes with ≤5% read-out and >35% read-out. Only genes with ≤5% read-in were considered in both cases.

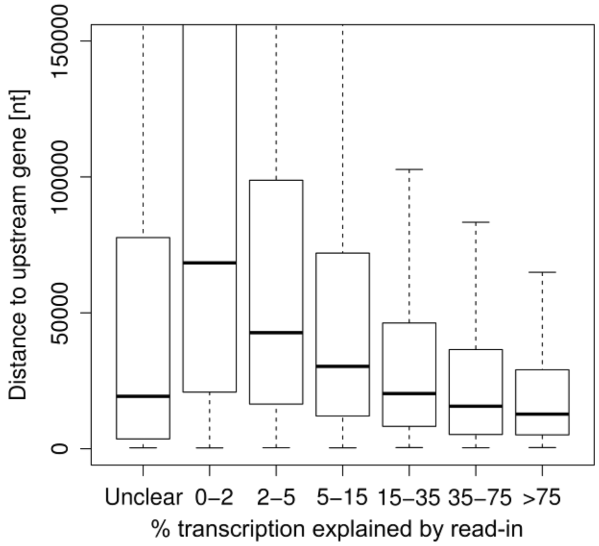

Supplementary Figure 6: Read-in is inversely correlated with the distance to the next upstream gene

The distribution of distances to the next upstream gene relative to the extent of read-in (=100 x RPKM upstream of gene/gene RPKM at 7-8h p.i.) is visualized using boxplots. The boxes indicate the range between the 25th and 75th percentile (=interquartile range [IQR]) around the median (thick horizontal line) of the distribution. The whiskers (=short horizontal lines at ends of dashed vertical line) extend to the data points at most 1.5 x IQR from the box. For clarity, the extreme data points outside the whiskers are not shown. The higher read-in levels, the closer the upstream gene tended to be, showing that the distance to the upstream gene largely determines the extent of read-in.

a

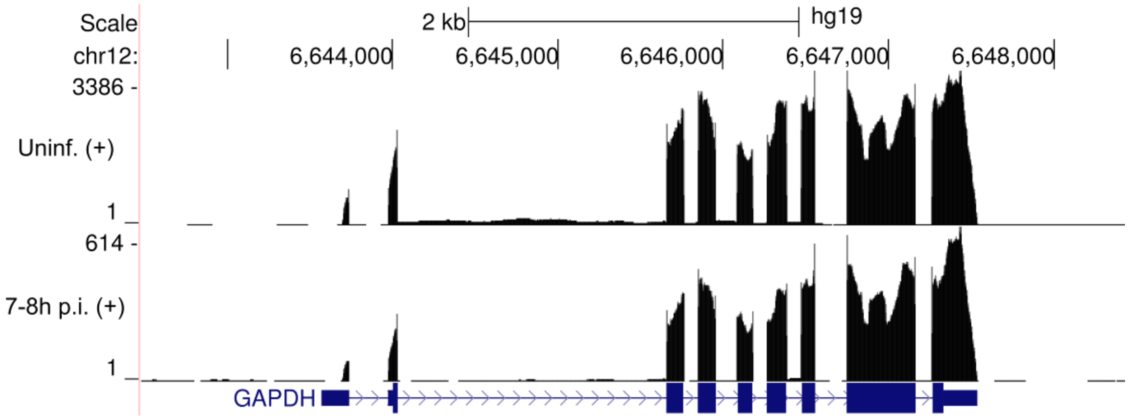

b

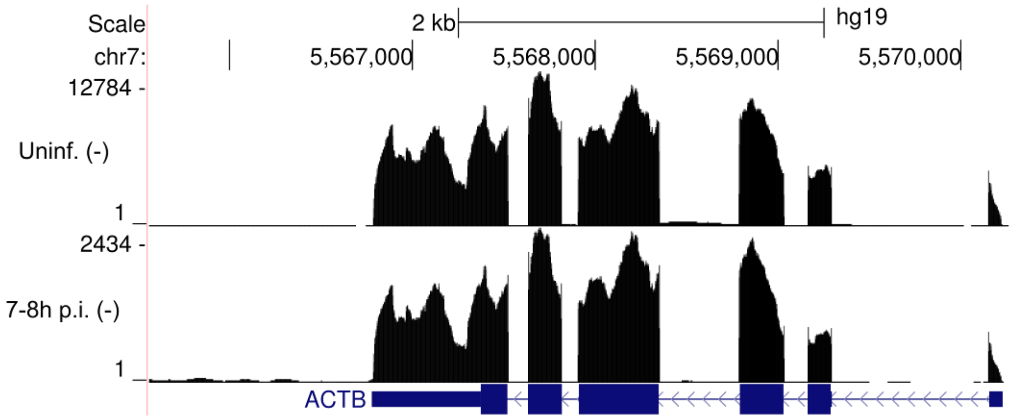

Supplementary Figure 7: Transcriptional activity for GAPDH and β-actin

Only very little transcription is observed in 4sU-RNA both upstream and downstream of the two housekeeping genes GAPDH (**a**) and β-actin (ACTB) (**b**), indicating that these are affected neither by read-in nor read-out. In addition, introns are still completely spliced for both genes at late stages of infection. Forward (+) and reverse (-) strands are indicated. Gene loci are depicted in blue as described in Fig. 4.

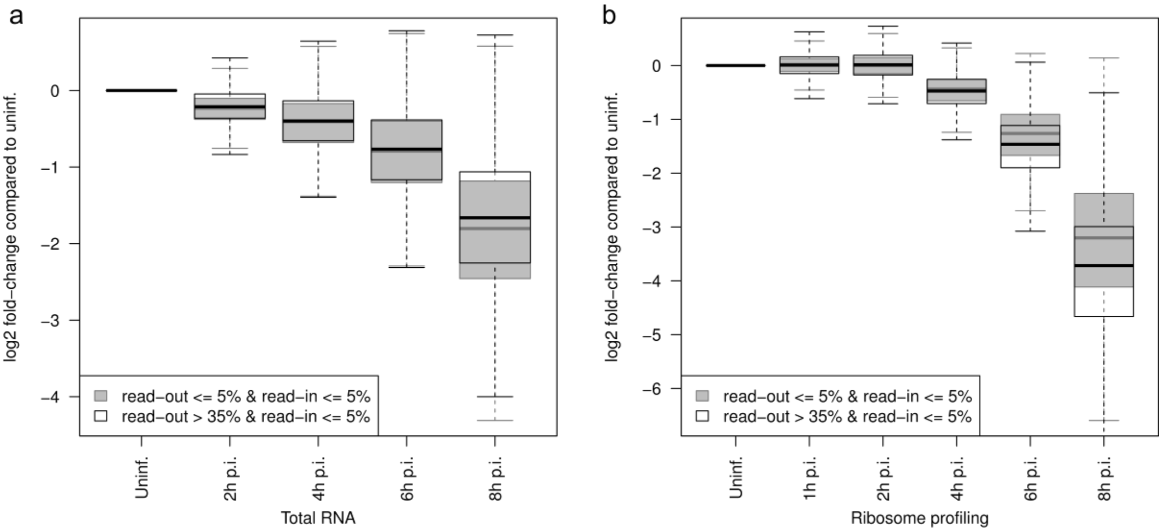

Supplementary Figure 8: Genes with high read-out show a stronger decrease in translational activity

Decrease in total RNA levels (a) and translational activity (b) was compared between genes with no or little (≤5%) read-out and genes with high (>35%) read-out in 7-8h p.i. 4sU-RNA. In both cases, only genes with ≤5% read-in were considered. At each time point, log2 fold-changes between the gene RPKM at this time point and the gene RPKM in uninfected cells were calculated for all genes and distribution of fold-changes were illustrated using boxplots as in Supplementary Figure 6. Genes with >35% read-out were on average not down-regulated more strongly in total RNA than genes without read-out (**a**). In contrast, their translation rates showed a higher reduction on average at 6h and 8h p.i. (**b,** Wilcox rank sum test $p < 10^{-8}$).

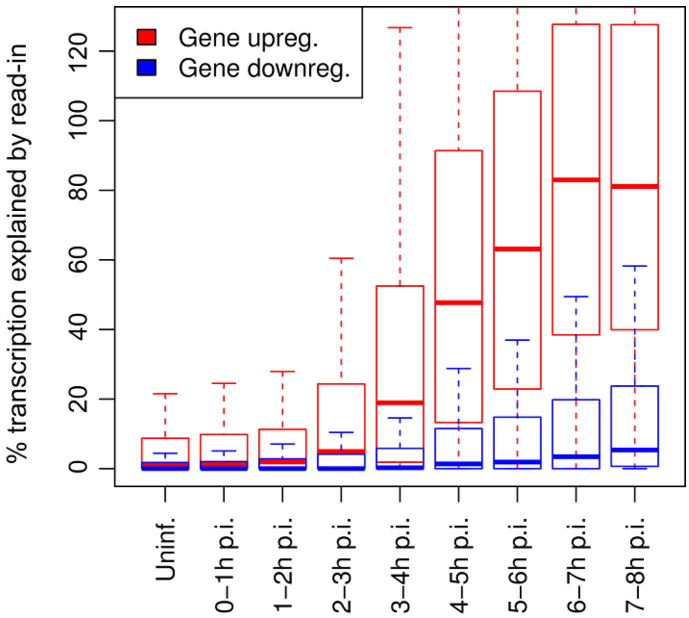

Supplementary Figure 9: Read-in is responsible for the seeming induction of genes

Extent of read-in in 7-8h p.i. 4sU-RNA was compared between genes up-regulated and down-regulated in 7-8h p.i. 4sU-RNA. As in Supplementary Figure 6, boxplots were used to visualize the distribution of the %transcription with read-in (quantified as 100 x RPKM upstream of gene/ gene RPKM in 7-8h p.i. 4sU-RNA) at each time point. Up-regulated genes show significantly more read-in than down-regulated genes.

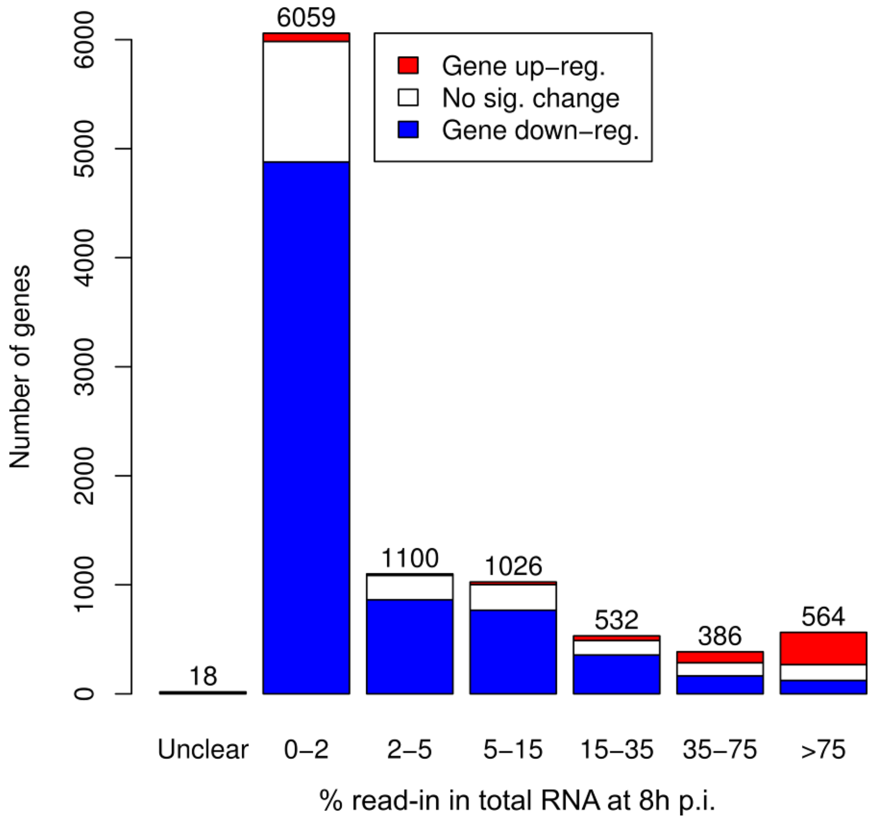

Supplementary Figure 10: Read-in is observed in total RNA and leads to apparent gene induction in total RNA

In this figure, number of genes with different extent of read-in in 8h p.i. total RNA are shown as well as the number of genes up- or down-regulated in total RNA at 8h p.i. Here, the extent of read-in was quantified as 100 x RPKM in the first 5,000nt upstream of the gene/gene RPKM (both in 8h p.i. total RNA). Genes with a high extent of read-in in total RNA were also enriched for genes induced in total RNA (≥2-fold, see Methods).

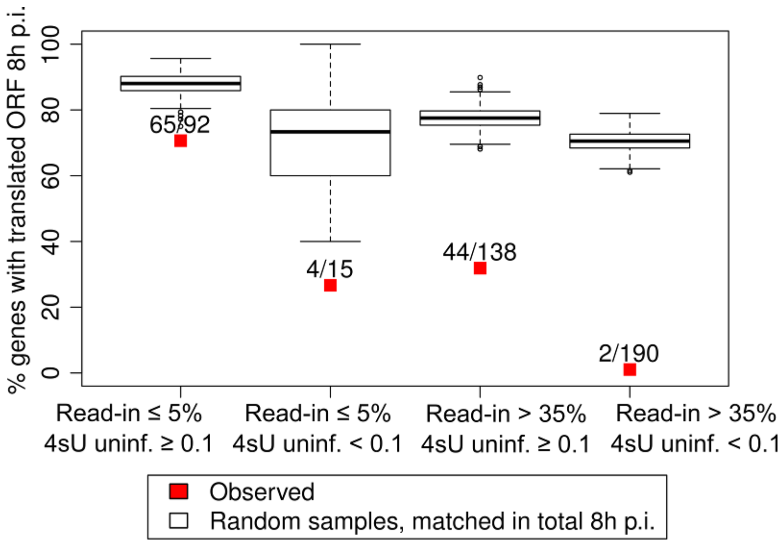

Supplementary Figure 11: Genes induced by read-in are not translated

The percentage of ORFs translated at 8h p.i. is shown for transcriptionally induced genes with and without read-in as well as genes either transcribed (RPKM≥0.1) or not transcribed (RPKM<0.1) in uninfected cells (4sU-RNA). The significance of the obtained results was evaluated by comparing the number of translated ORFs against 1,000 randomly sampled gene sets with non-induced genes matched in number and RPKM values (± 0.1) in total RNA 8h p.i. The distribution of the percentage of translated ORFs is visualized using boxplots as described in Supplementary Figure 6. The observed numbers of translated ORFs were significantly below the smallest values observed for any of the randomly sampled gene sets (p-value $<10^{-3}$) showing that the low translation rates for genes induced by read-in are not simply due to their low total RNA levels.

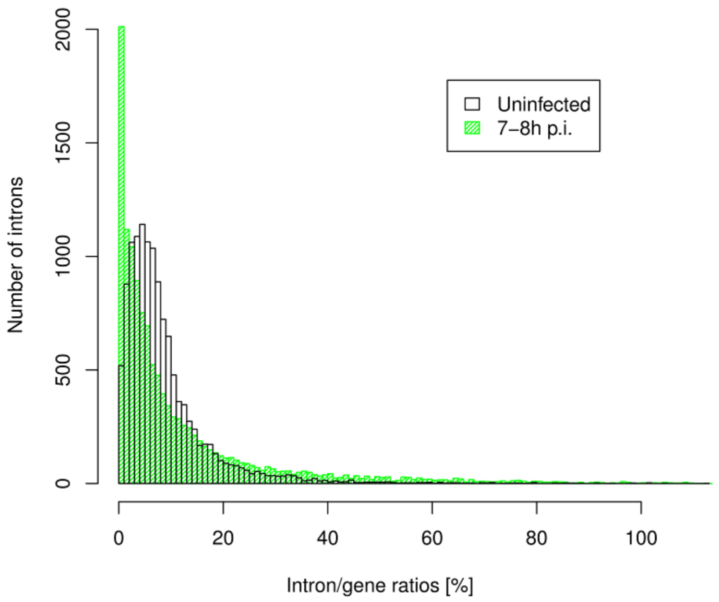

Supplementary Figure 12: Splicing is not generally inhibited during HSV-1 infection

For 12,498 introns (RPKM ≥1 in all 4sU-RNA samples) in ~2,000 highly expressed genes (RPKM ≥10 in uninfected 4sU-RNA), we compared the distribution of intron/gene ratios in uninfected cells and 7-8h p.i. Although the number of introns with high intron/gene ratios was slightly increased, there was no overall trend towards high intron/gene ratios at 7-8h p.i. and thus no genome-wide accumulation of unspliced pre-mRNAs.

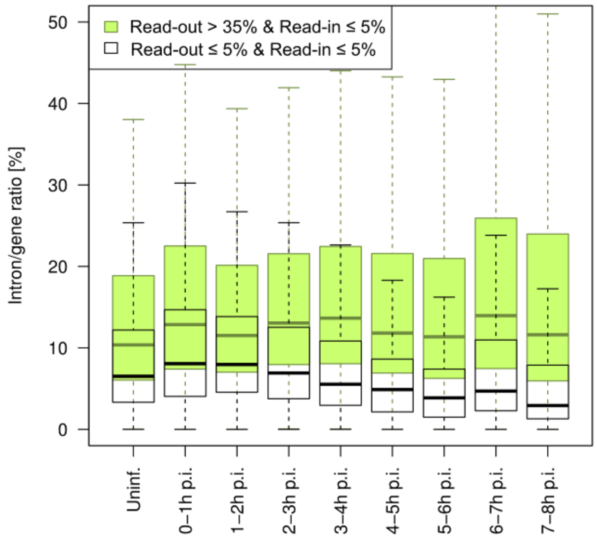

Supplementary Figure 13: Read-out does not lead to increased intron/gene ratios

Intron/gene ratios during the course of virus infection were compared between genes with high (>35%) and very low/no (≤5%) read-out but no read-in (≤5%). The distribution of intron/gene ratios (in %) for each time point is visualized using boxplots as in Supplementary Figure 6. Only down-regulated genes were included in this analysis. Intron/gene ratios did not increase during virus infection in either case.

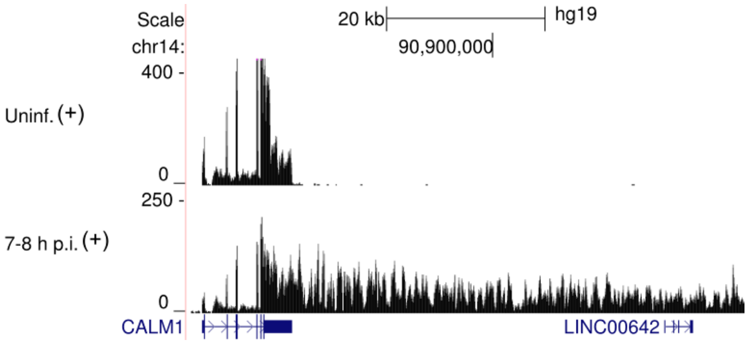

Supplementary Figure 14: Example of read-in into a lincRNA

Example of read-out from a well-spliced protein-coding gene (*CALM1*) into a subsequently unspliced lincRNA (*LINC00642*). Gene loci are depicted in blue as described in Fig. 4.

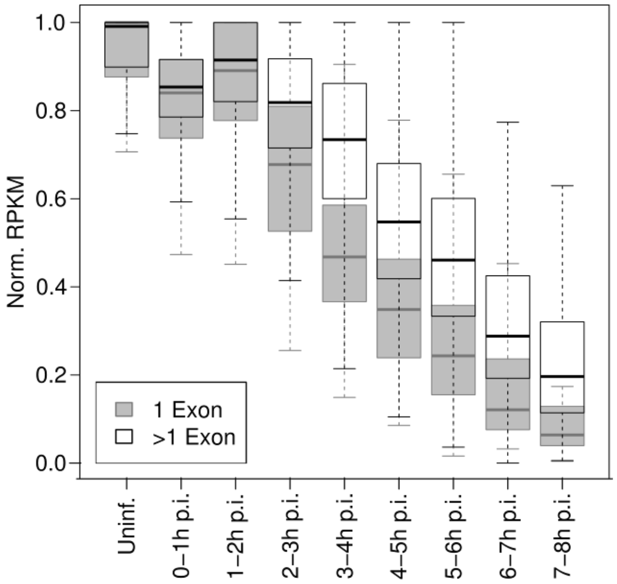

Supplementary Figure 15: Unspliced pre-mRNAs are not degraded more rapidly

For all down-regulated genes, RPKM values were normalized against their maximum RPKM value across all time points and compared between genes with and without introns. Distributions of normalized RPKM values in 4sU-RNA were visualized using boxplots as described in Supplementary Figure 6. This demonstrates that intron-containing genes are not down-regulated faster than single-exon, i.e. intron-less, genes. If unspliced or incorrectly spliced pre-mRNAs were rapidly degraded, 4sU-RNA levels of spliced, i.e. intron-containing genes, should decrease faster than RNA levels of single-exon genes.

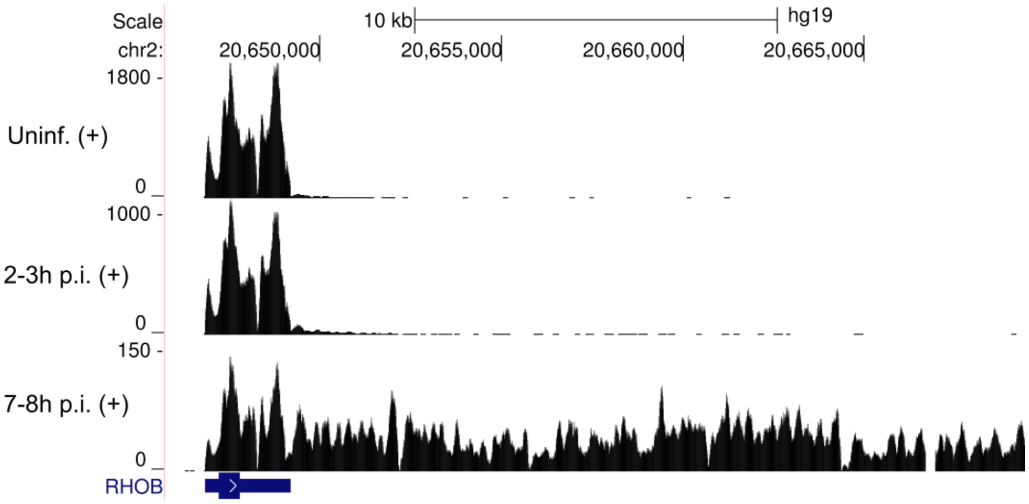

Supplementary Figure 16: Intron-less genes also show read-out

Example of a intron-less gene with read-out but no read-in on the forward strand (indicated by ‘+’). Gene loci including coding and untranslated sequences are depicted in blue as described in Fig. 4.

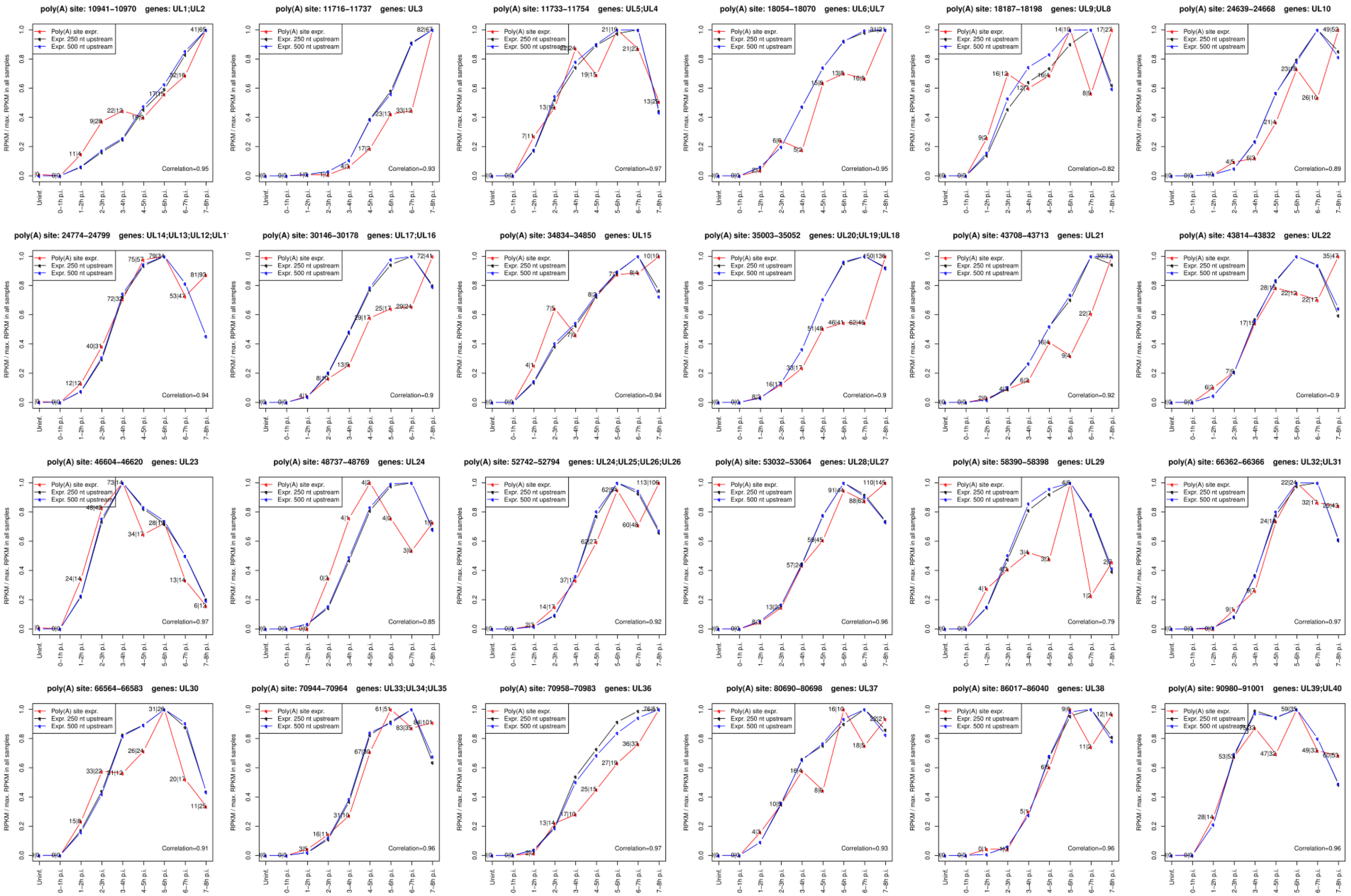
poly(A) site: 10941–10970 genes: UL1;UL2
Correlation=0.95
poly(A) site: 11716–11737 genes: UL3
Correlation=0.93
poly(A) site: 11733–11754 genes: UL5;UL4
Correlation=0.97
poly(A) site: 18054–18070 genes: UL6;UL7
Correlation=0.95
poly(A) site: 18187–18198 genes: UL9;UL8
Correlation=0.82
poly(A) site: 24639–24668 genes: UL10
Correlation=0.89
poly(A) site: 24774–24799 genes: UL14;UL13;UL12;UL1
Correlation=0.94
poly(A) site: 30146–30178 genes: UL17;UL16
Correlation=0.9
poly(A) site: 34834–34850 genes: UL15
Correlation=0.94
poly(A) site: 35003–35052 genes: UL20;UL19;UL18
Correlation=0.9
poly(A) site: 43708–43713 genes: UL21
Correlation=0.92
poly(A) site: 43814–43832 genes: UL22
Correlation=0.9
poly(A) site: 46604–46620 genes: UL23
Correlation=0.97
poly(A) site: 48737–48769 genes: UL24
Correlation=0.85
poly(A) site: 52742–52794 genes: UL24;UL25;UL26;UL26
Correlation=0.92
poly(A) site: 53032–53064 genes: UL28;UL27
Correlation=0.96
poly(A) site: 58390–58398 genes: UL29
Correlation=0.79
poly(A) site: 66362–66366 genes: UL32;UL31
Correlation=0.97
poly(A) site: 66564–66583 genes: UL30
Correlation=0.91
poly(A) site: 70944–70964 genes: UL33;UL34;UL35
Correlation=0.96
poly(A) site: 70958–70983 genes: UL36
Correlation=0.97
poly(A) site: 80690–80698 genes: UL37
Correlation=0.93
poly(A) site: 86017–86040 genes: UL38
Correlation=0.96
poly(A) site: 90980–91001 genes: UL39;UL40
Correlation=0.96
Poly(A) site expr.
Expr. 250 nt upstream
Expr. 500 nt upstream
RPKM / max. RPKM in all samples
Uninf.
0–1h p.i.
1–2h p.i.
2–3h p.i.
3–4h p.i.
4–5h p.i.
5–6h p.i.
6–7h p.i.
7–8h p.i.

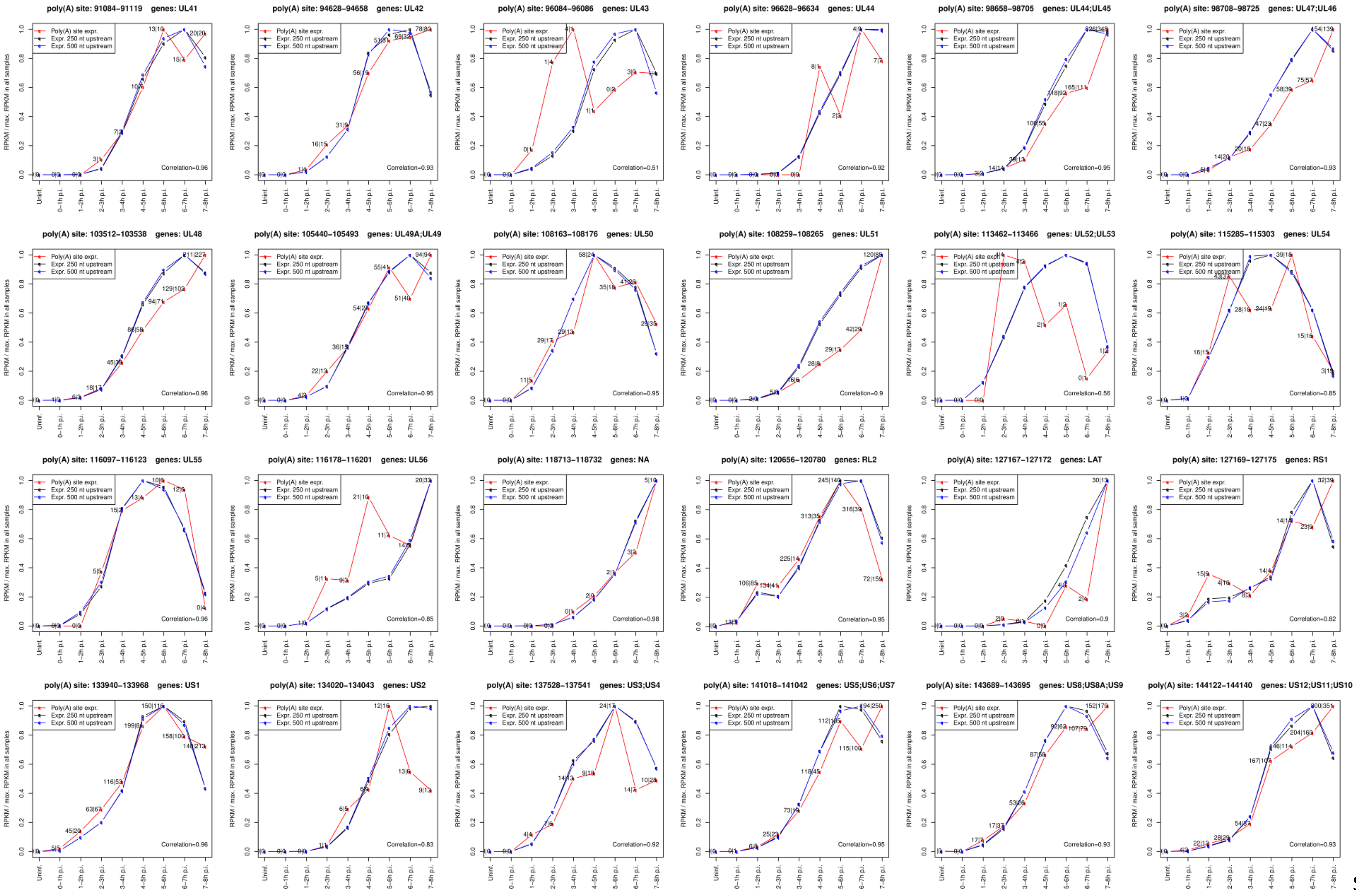
poly(A) site: 91084–91119 genes: UL41
poly(A) site: 94628–94658 genes: UL42
poly(A) site: 96084–96086 genes: UL43
poly(A) site: 96628–96634 genes: UL44
poly(A) site: 98658–98705 genes: UL44;UL45
poly(A) site: 98708–98725 genes: UL47;UL46
poly(A) site: 103512–103538 genes: UL48
poly(A) site: 105440–105493 genes: UL49A;UL49
poly(A) site: 108163–108176 genes: UL50
poly(A) site: 108259–108265 genes: UL51
poly(A) site: 113462–113466 genes: UL52;UL53
poly(A) site: 115285–115303 genes: UL54
poly(A) site: 116097–116123 genes: UL55
poly(A) site: 116178–116201 genes: UL56
poly(A) site: 118713–118732 genes: NA
poly(A) site: 120656–120780 genes: RL2
poly(A) site: 127167–127172 genes: LAT
poly(A) site: 127169–127175 genes: RS1
poly(A) site: 133940–133968 genes: US1
poly(A) site: 134020–134043 genes: US2
poly(A) site: 137528–137541 genes: US3;US4
poly(A) site: 141018–141042 genes: US5;US6;US7
poly(A) site: 143689–143695 genes: US8;US8A;US9
poly(A) site: 144122–144140 genes: US12;US11;US10
Poly(A) site expr.
Expr. 250 nt upstream
Expr. 500 nt upstream
RPKM / max. RPKM in all samples
Uninf. 0–1h p.i. 1–2h p.i. 2–3h p.i. 3–4h p.i. 4–5h p.i. 5–6h p.i. 6–7h p.i. 7–8h p.i.
Correlation=0.96
Correlation=0.93
Correlation=0.51
Correlation=0.92
Correlation=0.95
Correlation=0.93
Correlation=0.96
Correlation=0.95
Correlation=0.95
Correlation=0.9
Correlation=0.56
Correlation=0.85
Correlation=0.96
Correlation=0.85
Correlation=0.98
Correlation=0.95
Correlation=0.9
Correlation=0.82
Correlation=0.96
Correlation=0.83
Correlation=0.92
Correlation=0.95
Correlation=0.93
Correlation=0.93

Su

Supplementary Figure 17: Poly(A) sites in HSV-1

Poly(A) sites in the HSV-1 genome were identified by mapping 4sU-RNA reads containing part of the poly(A) sequence (see Methods for details). For all identified poly(A) sites, we correlated changes in RPKM within 250 and 500nt upstream of the poly(A) site, respectively, with the changes in the number of reads mapping to this poly(A) site [= poly(A) site expression] during infection. Here, changes are visualized by normalizing all RPKM values to the maximum RPKM across all time-points. Number of reads mapping to reads with part of a poly(A) tail in each replicate are annotated to the curve for poly(A) site expression (separated by '|'). Position of the poly(A) site and corresponding genes are indicated above each curve and correlation between expression 500nt upstream and poly(A) site expression is shown in the bottom-right corner.

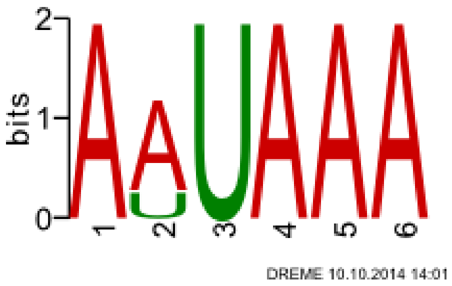

Supplementary Figure 18: Poly(A) signal motif

This Figure shows the motif identified using the DREME (Discriminative DNA Motif Discovery) tool in the MEME motif search suite (see main manuscript for references) within the 50nt upstream of gene 3'-ends. This corresponds to two most frequent poly(A) signal sequences previously reported.
